# Phosphoproteomics identification of ERK-dependent activation of Rps6kb1 in cardiac hypertrophy

**DOI:** 10.1172/jci.insight.190760

**Published:** 2026-01-08

**Authors:** Chao Li, Pengfei Zhang, Kai Zhang, Jane A. Cook, Weidan Song, Megan Virostek, Lily A. Slotabec, Nadiyeh Rouhi, Mohammed Hazari, Michael I. Adenawoola, Xiaofei Liu, Hao Zhang, Guangyu Zhang, Erica L. Niewold, Qinfeng Li, Yong Fang, Waleed M. Elhelaly, Xue-Nan Sun, Xuejiang Guo, Andrew Lemoff, Yingfeng Deng, Thomas G. Gillette, Ji Li, Philipp E. Scherer, Zhao V. Wang

**Affiliations:** 1 Division of Cardiology, and; 2 Touchstone Diabetes Center, Department of Internal Medicine, The University of Texas Southwestern Medical Center, Dallas, Texas, USA.; 3 Department of Diabetes and Cancer Metabolism, Beckman Research Institute, City of Hope National Medical Center, Duarte, California, USA.; 4 Department of Physiology and Biophysics, University of Mississippi Medical Center, Jackson, Mississippi, USA.; 5 State Key Laboratory of Reproductive Medicine and Offspring Health, Department of Histology and Embryology, Nanjing Medical University, Nanjing, China.; 6 Department of Biochemistry, The University of Texas Southwestern Medical Center, Dallas, Texas, USA.; 7 G.V. (Sonny) Montgomery VA Medical Center, Jackson, Mississippi, USA.

**Keywords:** Cardiology, Cell biology, Cardiovascular disease, Heart failure, Molecular biology

## Abstract

Cardiomyocyte growth is tightly controlled by multiple signaling pathways. Identification of master kinases in this process is essential in exploring potential targets for the treatment of pathological cardiac hypertrophy and heart failure. Here we identified the mTOR-independent activation of ribosomal protein S6 kinase b1 (Rps6kb1) during cardiomyocyte growth. By utilizing phosphoproteomics in primary neonatal rat ventricular myocytes, we revealed Rps6kb1 as one of most activated kinases under growth stimulation. We further demonstrated the role of Rps6kb1 phosphorylation in pathological cardiac hypertrophy and heart failure. We showed that the phosphorylation of multiple sites in Rps6kb1, including T367 in the kinase domain and S418/T421/S424 in the C-terminal domain, is not directly regulated by the activity of mTOR but coupled with the activation of the MEK1/ERK axis. In mice, cardiomyocyte-specific deletion of Rps6kb1 significantly inhibited both constitutively active ERK– and pressure overload–induced cardiac hypertrophy. In contrast, cardiomyocyte-specific overexpression of wild-type Rps6kb1, rather than the phosphorylation-defective mutant, elevated cardiac hypertrophy and augmented pressure overload–induced heart failure. In conclusion, our findings reveal that the MEK/ERK axis primes Rps6kb1 activation through phosphorylation of 2 separate domains of Rps6kb1, which may play an essential role in cardiac hypertrophy and heart failure under hemodynamic stress.

## Abstract 2

Li and Zhang et al. show that ERK-dependent phosphorylation of Rps6kb1 plays an essential role in cardiac hypertrophic growth.

## Introduction

Heart failure is a leading cause of death worldwide (1). Heart-related diseases, including hypertensive heart disease and myocardial infarction, are inevitably associated with the growth of cardiomyocytes, a process also known as cardiac hypertrophy. Under persistent stress, this once adaptive response may decompensate and succumb to heart failure (2). Despite extensive clinical relevance, our understanding of this pathological process remains incomplete (3, 4).

Cardiac hypertrophic growth is a tightly controlled process, in which protein kinases play a pivotal role in relaying cellular signals (5). Among all kinases, mTOR is considered a master regulator that senses divergent environmental cues and controls key cellular processes, including protein synthesis (6). In addition to a fundamental role of mTOR in cardiogenesis (7, 8), genetic disruption of mTOR in adult hearts manifests heightened development of heart failure under pressure overload (9, 10). Nevertheless, the direct downstream targets of mTOR signaling during pathological cardiac remodeling are incompletely understood. Ribosomal protein S6 kinase b1 (Rps6kb1, also known as S6K1), whose phosphorylation at T389 is a hallmark of mTORC1 activity, is probably the most promising candidate, considering its role in protein synthesis (11, 12). However, our understanding of Rps6kb1 in cardiac hypertrophic growth and pathological remodeling remains obscure (13).

Here, through phosphoproteomics analysis in cultured cardiomyocytes, we demonstrate that Rps6kb1 is one of the most prominent kinases during hypertrophic growth. We show that Rps6kb1 is activated by the MEK/ERK axis. We go on to identify 4 residues of Rps6kb1 that are directly phosphorylated by ERK. Furthermore, cardiomyocyte-specific Rps6kb1 deletion in mice almost completely abolished cardiac hypertrophy induced by either ERK activation or pressure overload. Importantly, overexpression of wild-type (WT) Rps6kb1, rather than phosphorylation-defective Rps6kb1, drove cardiac hypertrophic growth. These findings together demonstrate a critical role of the ERK/Rps6kb1 axis in cardiac hypertrophic growth, which may represent a promising target for the treatment of hypertensive heart disease and heart failure.

## Results

### mTOR-independent phosphorylation of Rps6kb1 is increased in cardiomyocytes subjected to growth stimulation.

**Figure 1:**
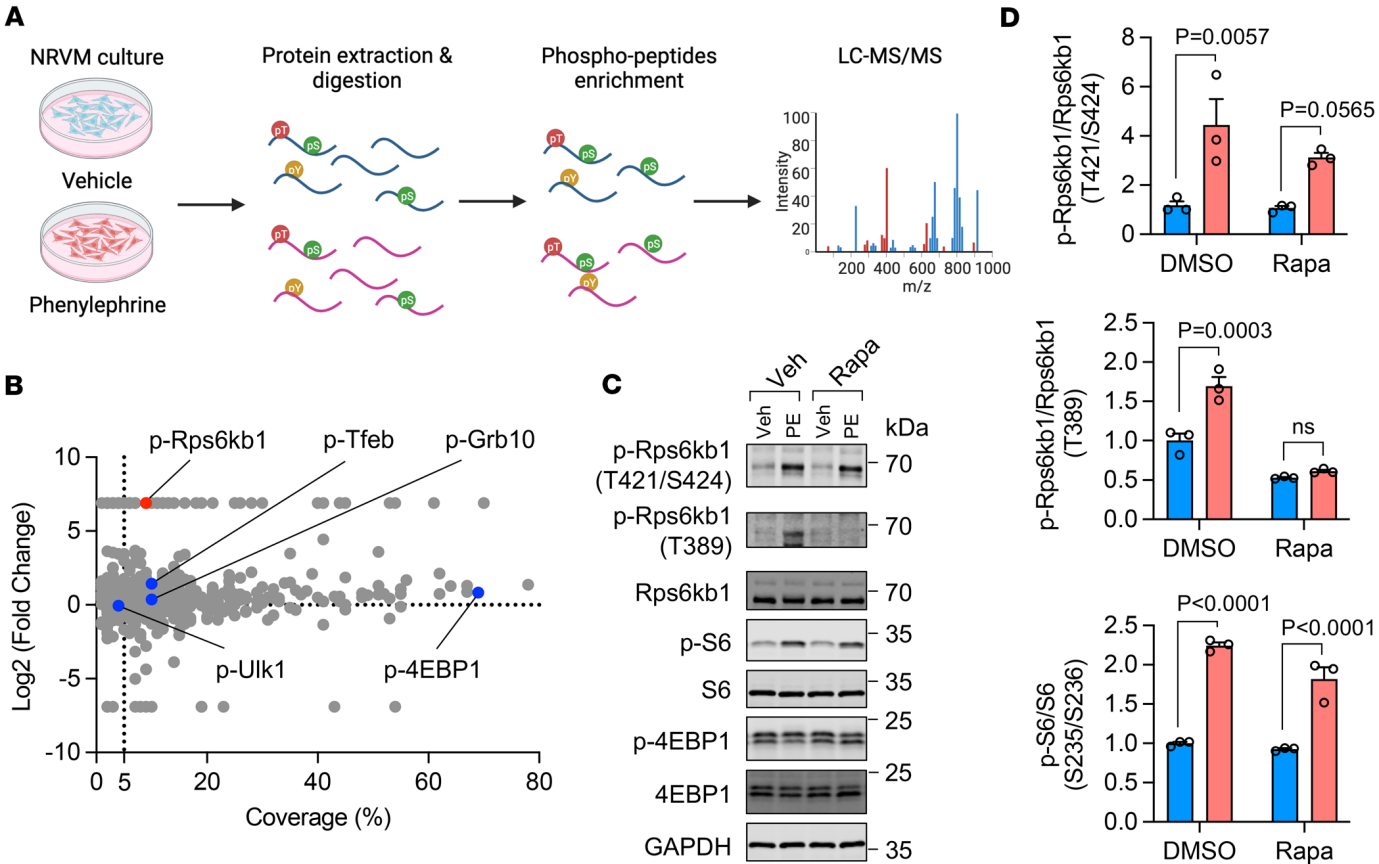
The mTOR-independent phosphorylation of Rps6kb1 in cardiomyocytes under hypertrophic growth. (**A**) Schematic diagram of phosphoproteomics assay. Neonatal rat ventricular myocytes (NRVMs) were isolated and treated with or without phenylephrine (PE) for 30 minutes. (**B**) Analysis for all phosphorylated proteins identified in **A**. Several mTORC1 targets are labeled, including Rps6kb1, Tfeb, Ulk1, Grb10, and 4EBP1. (**C**) Rapamycin (Rapa) inhibited the phosphorylation of Rps6kb1 at T389 but not the T421/S424 sites. NRVMs were stimulated by PE for 15 minutes with or without cotreatment with Rapa. Note that PE and Rapa were simultaneously added into culture medium. (**D**) Quantification of the results in **C**. *n* = 3. NS, not significant. Two-way ANOVA was conducted, followed by Tukey’s multiple-comparison test for **D**. Data are presented as mean ± SEM.

To explore the kinase network during cardiomyocyte hypertrophic growth, we employed an unbiased phosphoproteomics approach (Figure 1A). We treated primary neonatal rat ventricular myocytes (NRVMs) with phenylephrine (PE), a commonly used method to model cardiomyocyte hypertrophy in vitro (14). We extracted total proteins and enriched phosphorylated peptides. We then identified the sequences and modifications of peptides by liquid chromatography with tandem mass spectrometry (LC-MS/MS) (Supplemental Tables 1–9; supplemental material available online with this article; https://doi.org/10.1172/jci.insight.190760DS1). Rps6kb1 was one of the most prominent kinases with the highest enrichment when comparing PE with vehicle treatments (Figure 1B), suggesting that Rps6kb1 may play a pivotal role in cardiomyocyte hypertrophic growth.

Rps6kb1 is an established downstream kinase of mTOR complex 1 (mTORC1), a master regulator of cell growth (6). In addition to mTORC1, Rps6kb1 is also phosphorylated by other kinases under different physiological or pathophysiological conditions (15). Nevertheless, the role of Rps6kb1 and its activating kinase(s) remain to be fully illustrated in cardiac hypertrophy. In addition to Rps6kb1, mTORC1 also directly phosphorylates other targets, such as 4EBP1, Ulk1, Tfeb, and Grb10. Importantly, we found that the enrichment of these targets of mTORC1 was less than that for Rps6kb1 (Figure 1B), indicating that mTORC1 activity may not fully account for the drastic elevation of Rps6kb1 phosphorylation under cardiomyocyte growth stimulation.

To further evaluate the change in Rps6kb1 phosphorylation after PE treatment, we employed 2 antibodies, one recognizing the mTORC1-dependent phosphorylation at T389 and the other one for the phosphorylation of T421 and S424 sites in the C-terminal domain (CTD) of Rps6kb1 whose upstream kinase remains unidentified. We found that the phosphorylation of T421/S424 was significantly increased upon PE treatment, as well as the mTOR-dependent phosphorylation of T389 (Figure 1, C and D). Consistent with the phosphoproteomics results, phosphorylation of 4EBP1 was not increased, indicating moderate activation of mTORC1. Importantly, simultaneous rapamycin treatment with PE completely abolished the induction of T389 phosphorylation of Rps6kb1 but had minimal effect on T421/S424 phosphorylation (Figure 1, C and D). Moreover, the activity of Rps6kb1 in NRVMs, manifested by the phosphorylation of S6, was activated regardless of rapamycin treatment. These findings prompted us to hypothesize that an unrecognized upstream kinase, independent from mTORC1, may phosphorylate and activate Rps6kb1 during cardiomyocyte hypertrophic growth.

### Activation of the MEK/ERK axis is coupled with Rps6kb1 phosphorylation.

**Figure 2:**
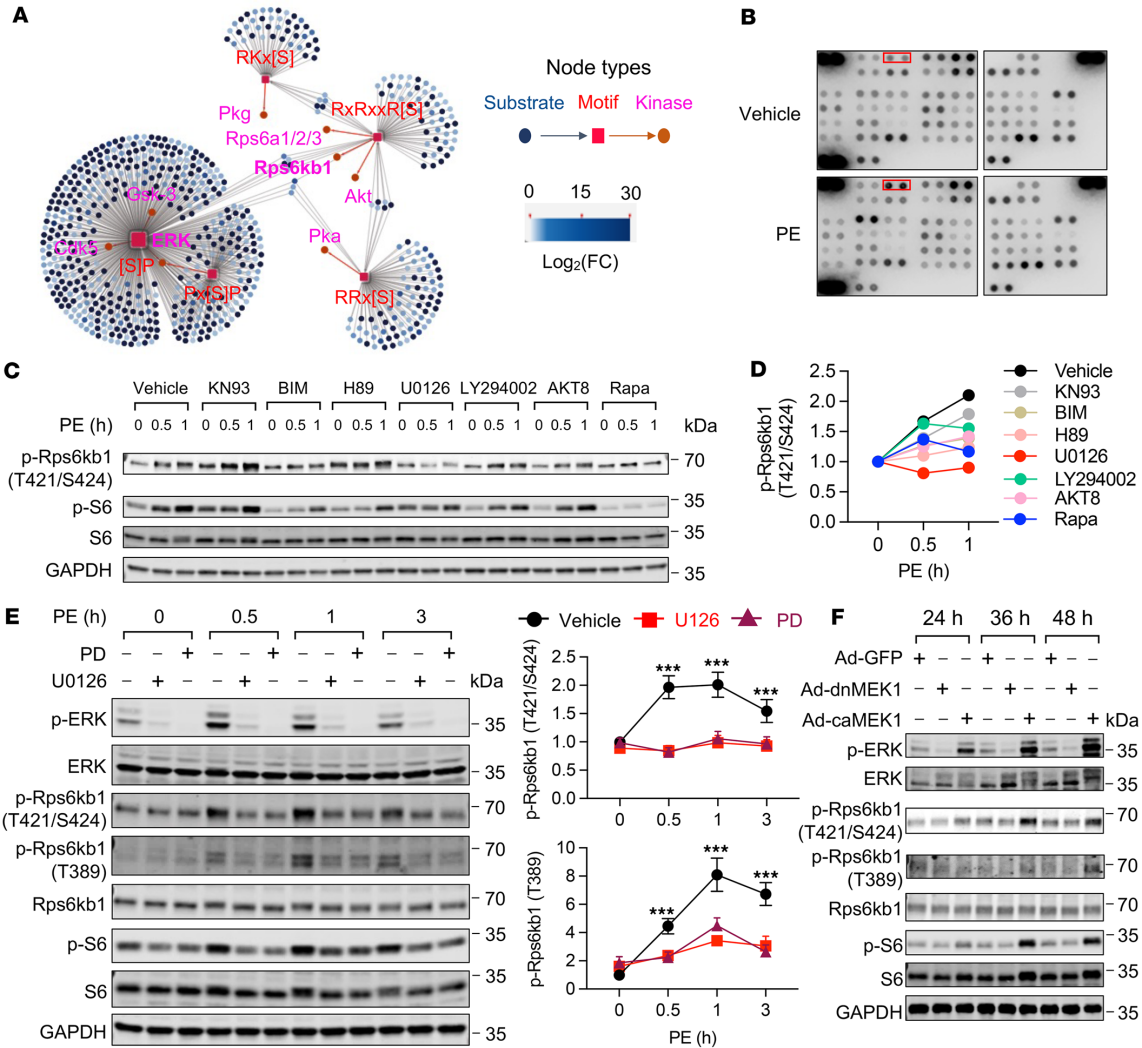
Phosphorylation of Rps6kb1 is coupled with the activation of ERK. (**A**) Motif enrichment analysis for the phosphorylated peptides identified in Figure 1A. Five motifs were enriched, including [S]P, Px[S]P, RKx[S], RxRxxR[S], and RRx[S]. (**B**) Phospho-kinase array was performed using NRVMs treated with PE for 30 minutes. Total cell lysates were extracted to hybridize phospho-kinase array membranes. The signal of p-ERK is boxed in red. (**C**) Seven kinase inhibitors were used to identify the kinase responsible for the phosphorylation of Rps6kb1 at the T421/S424 sites under PE treatment. Note that the inhibitors were added to the culture medium 1 hour before PE treatment. (**D**) Quantification for p-Rps6kb1 (T421/S424) shown in **C**. Only ERK inhibitor U0126 completely repressed the phosphorylation of Rps6kb1 induced by PE. (**E**) Inhibition of the MEK/ERK axis completely abolished the phosphorylation of Rps6kb1 at T421/S424 but not T389. NRVMs were treated with PE for 0.5, 1, and 3 hours, with or without cotreatment with MEK1/2 inhibitor U0126 or PD0325901. Note that the inhibitors were added into the culture medium 1 hour before PE treatment. *n* = 3. ***P < 0.0001 comparing vehicle with either U0126 or PD0325901 treatment. (**F**) ERK was selectively activated and repressed by constitutively active and dominant-negative MEK1 (caMEK1 and dnMEK1), respectively. NRVMs were infected by adenoviruses for 24, 36, or 48 hours. Western blotting was conducted to examine the phosphorylation of ERK, Rps6kb1, and S6. One-way ANOVA was conducted, followed by Tukey’s multiple-comparison test for **E**. Data are presented as mean ± SEM.

We next sought to identify the kinase that is responsible for the mTOR-independent phosphorylation of Rps6kb1. In the phosphoproteomics data, we extracted phosphorylated peptides and identified a total of 8,639 sites, among which 1,383 sites were never reported (Supplemental Figure 1, A and B). We then performed motif enrichment analysis for peptides that had significant differences between vehicle and PE groups. We identified 5 motifs with statistical significance (Figure 2A and Supplemental Figure 1C). We then predicted potential kinases for these motifs. Importantly, ERK showed the most abundant phosphorylation targets under PE treatment in cardiomyocytes (Figure 2A). Next, a phospho-kinase array assay was conducted comparing PE to vehicle treatments. Phosphorylation of ERK1/2 was also increased in cardiomyocytes (Figure 2B). We further examined the effect of various kinase inhibitors on the phosphorylation of Rps6kb1 at T421/S424 (Figure 2, C and D, and Supplemental Figure 1D), which again pointed to ERK. Taken together, these results suggest that ERK may be in the upstream kinase of Rps6kb1 for T421/S424 phosphorylation under hypertrophic growth in cardiomyocytes.

To further address whether the activity of the MEK/ERK axis is coupled to the mTOR-independent phosphorylation of Rps6kb1, we applied 2 MEK1/2 inhibitors in NRVMs. Phosphorylation of Rps6kb1 at T421/S424 was abolished with MEK1/2 inhibitors after PE treatment, while mTOR-dependent T389 phosphorylation was still induced by PE, albeit repressed to a certain degree (Figure 2E). The basal level of Rps6kb1 phosphorylation at T421/S424 under the treatment with MEK1/2 inhibitors may be due to other signaling pathways other than MEK/ERK. To manipulate the MEK/ERK axis, we infected NRVMs with adenovirus expressing constitutively active MEK1 (caMEK1) and dominant-negative MEK1 (dnMEK1), which selectively activate and repress ERK, respectively. We found that caMEK1 increased, whereas dnMEK1 inhibited, the phosphorylation of Rps6kb1 at T421/S424 (Figure 2F). Collectively, these results strongly indicate that the MEK/ERK axis is coupled with mTOR-independent phosphorylation of Rps6kb1.

### Rps6kb1 is required for ERK-indued cardiomyocyte growth.

**Figure 3:**
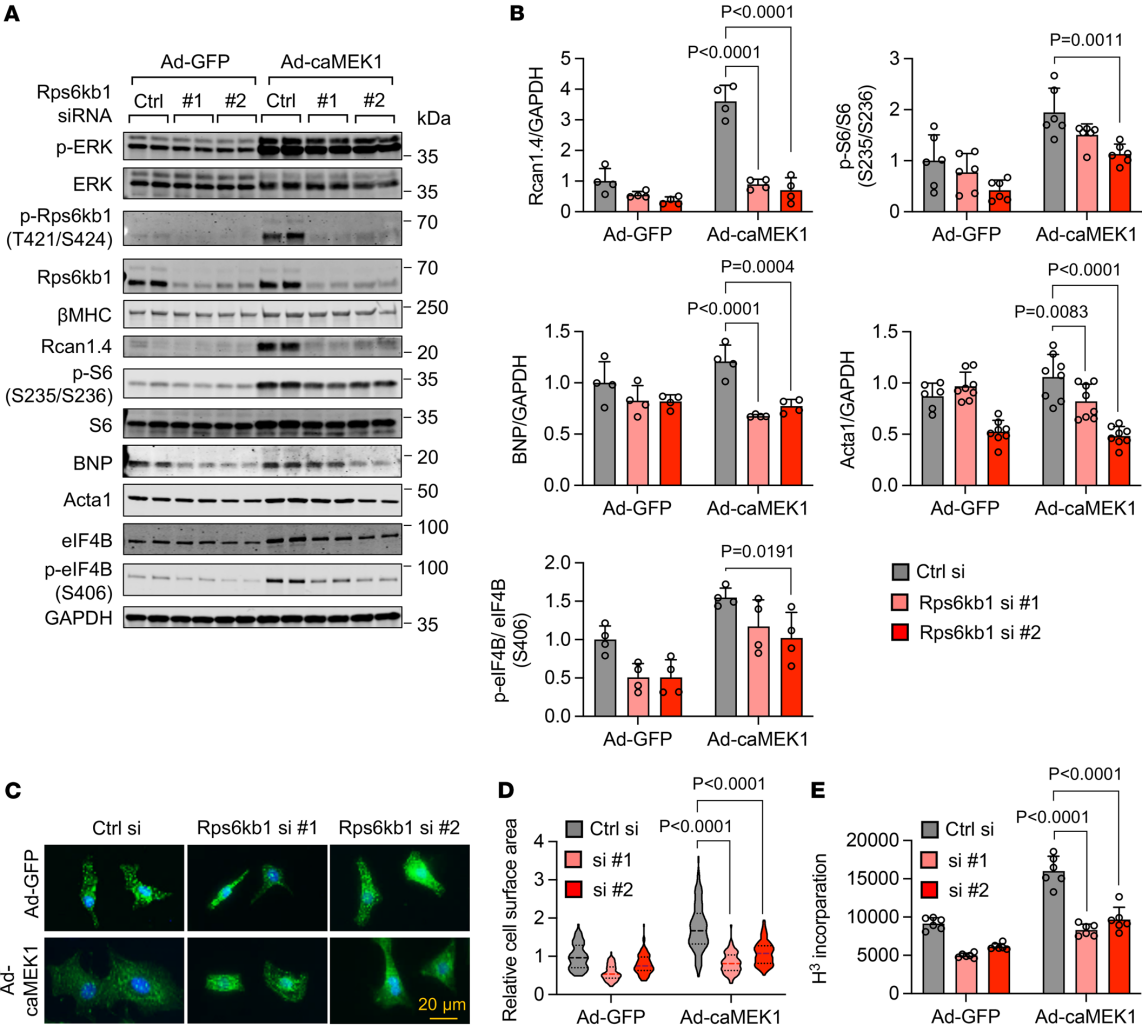
Rps6kb1 is required for ERK-induced cardiomyocyte growth. (**A**) Rps6kb1 silencing reduced the expression of marker genes related to cardiomyocyte growth at the protein level. Two independent siRNAs against Rps6kb1 were used. NRVMs were infected with Ad-caMEK1 to induce hypertrophy. Western blotting was conducted to examine the phosphorylation of ERK, Rps6kb1, eIF4B, and S6 and the expression of Rcan1.4. BNP, and Acta1. (**B**) Quantification of **A**. *n* = 4–8. (**C**) Rps6kb1 silencing inhibited ERK-induced cardiomyocyte growth. NRVMs were cultured in serum-free medium. Immunofluorescent staining for α-actinin was conducted. Scale bar: 20 μm. (**D**) Quantification of cardiomyocyte surface area from **C**. Two independent siRNAs against Rps6kb1 were used. A total of 80–100 cardiomyocytes was quantified for individual groups. (**E**) Rps6kb1 knockdown decreased protein synthesis in NRVMs, as assayed by ^3^H-leucine incorporation. *n* = 6. Two-way ANOVA was conducted, followed by Tukey’s multiple-comparison test for **B**, **D**, and **E**. Data are presented as mean ± SEM.

We next asked whether Rps6kb1 is required for ERK-induced cardiomyocyte hypertrophic growth. Adenovirus-mediated caMEK1 overexpression significantly increased the phosphorylation of Rps6kb1 at T421/S424, phosphorylation of 2 Rps6kb1 targets (S6 and eIF4B), and protein levels of 3 cardiomyocyte hypertrophy markers (Acta1, BNP, and Rcan1.4) (Figure 3, A and B). Importantly, Rps6kb1 knockdown by siRNA significantly suppressed this trend. Similarly, under PE stimulation Rps6kb1 silencing significantly reduced the phosphorylation of S6 and eIF4B and expression levels of cardiomyocyte hypertrophy markers (Supplemental Figure 2, A and B). In addition, a previous report showed that Rps6ka1 (Rsk) also contributes to the phosphorylation of S6 (16), which may explain its partial downregulation upon Rps6kb1 silencing. We next conducted immunofluorescent staining for α-actinin (Figure 3C). The increase in cardiomyocyte surface area by caMEK1 was repressed by Rps6kb1 knockdown (Figure 3D). Consistently, the induction of protein synthesis by caMEK1 was diminished by Rps6kb1 silencing in NRVMs, as evaluated by ^3^H-leucine incorporation (Figure 3E). These findings together demonstrate that Rps6kb1 is indispensable for ERK-induced cardiomyocyte growth.

### Rps6kb1 is essential for ERK-induced cardiac hypertrophic growth in vivo.

**Figure 4:**
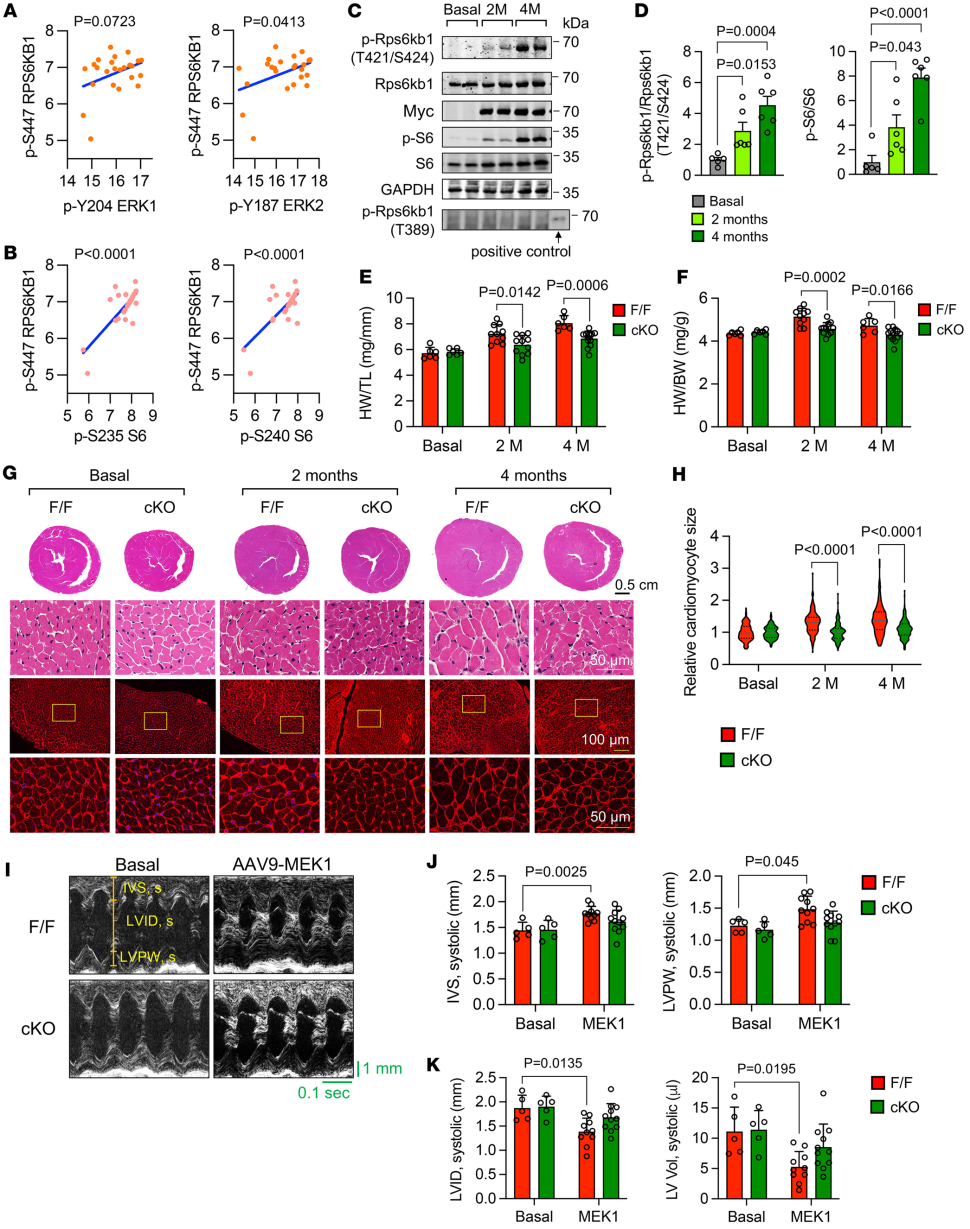
Rps6kb1 is essential for ERK-induced cardiac hypertrophy in vivo. (**A**) Correlation analysis for the phosphorylation of S447 of RPS6KB1 and ERK1/2 in heart samples from 24 patients with hypertrophic cardiomyopathy (HCM). The values were generated by phosphoproteomics from a previous report (17). Note that the S447 site of human RPS6KB1 corresponds to the S424 site of mouse Rps6kb1. (**B**) Correlation analysis for the phosphorylation of S447 of RPS6KB1 and S6 in heart samples from 24 patients with HCM. (**C**) Western blotting analysis for the heart tissues from control and caMEK1-overexpressing mice. Note that a positive control for T389 phosphorylation of Rps6kb1 was used. (**D**) Quantification of the phosphorylation of Rps6kb1 (T421/S424) and S6 in **C**. *n* = 5–6. (**E**) AAV9-caMEK1 induced cardiac hypertrophic growth in mice, which was significantly decreased under Rps6kb1 cardiac-specific deletion, as revealed by a reduction in the heart weight/tibia length (HW/TL) ratio. *n* = 6–13. (**F**) The heart weight/body weight (HW/BW) ratio was reduced in Rps6kb1-cKO mice. *n* = 6–13. (**G**) Representative histological images for heart sections from Rps6kb1^fl/fl^ control and Rps6kb1-cKO mice without or with myc-ERK2-MEK1 overexpression. After ERK2 was expressed for 2 or 4 months, cardiac tissues were harvested for H&E (upper) and wheat germ agglutinin (WGA, bottom) staining, respectively. Scale bars: 50 μm (top and bottom rows) and 100 μm (middle row). (**H**) Cardiomyocyte cross-sectional area was decreased in Rps6kb1-cKO hearts compared with Rps6kb1^fl/fl^ control hearts. A total of 128–167 cardiomyocytes was quantified from WGA staining in **G**. (**I**) Representative cardiac echocardiographic images. Note that IVS, LVID, and LVPW were quantified. (**J**) The thickness of septum and posterior wall of left ventricle, represented by IVS and IVPW, respectively, was attenuated in Rps6kb1-cKO mice after ERK2 overexpression for 2 months. *n* = 5–11. (**K**) The volume of ventricular chamber, represented by LVID and LV volume, was elevated in cKO mice after ERK2 overexpression for 2 months compared with controls. *n* = 5–11. Unpaired, 2-tailed Student’s *t* test was conducted for **D**. Two-way ANOVA was conducted, followed by Tukey’s multiple-comparison test for **E**, **F**, **H**, **J**, and **K**. Data are presented as mean ± SEM.

We went on to evaluate the role of the ERK/Rps6kb1 signaling in cardiac hypertrophic growth in vivo. We first assessed the correlation between the activity of ERK and RPS6KB1 in human hearts with hypertrophic cardiomyopathy (HCM). Recently, Garmany et al. explored the multiomics architecture of hypertrophic cardiomyopathy with flash-frozen cardiac tissues from patients with HCM undergoing surgical myectomy (17). Consistent with our findings, the Ras/ERK cascade was the most prominent signaling pathway activated by cardiac hypertrophy in humans (17). Importantly, in the phosphoproteomics dataset of 24 patients with HCM, the phosphorylation of RPS6KB1 at S447 in human hearts (corresponding to the phosphorylation at S424 of mouse Rps6kb1), but not the mTOR-dependent phosphorylation at T389, was detected. Moreover, there was a positive correlation between the phosphorylation of S447 of RPS6KB1 and ERK1/2 in 24 human hearts with HCM (Figure 4A). As a positive control, the phosphorylation of S447 of RPS6KB1 and S6 also showed a significant correlation in these samples (Figure 4B).

In mice, we overexpressed the constitutively active ERK2-MEK1 fused genes (18) in cardiomyocytes by adeno-associated virus 9 (AAV9) infection (Supplemental Figure 3, A and B). We found that overexpression of active ERK2 for 2 or 4 months significantly increased the phosphorylation of Rps6kb1 at T421/S424 in heart tissues but not the mTOR-dependent phosphorylation at T389 (Figure 4, C and D, and Supplemental Figure 3C). Phosphorylation of S6 was also elevated, indicating an increased activity of Rps6kb1 in the heart (Figure 4, C and D). Taken together, these findings suggest that the phosphorylation of Rps6kb1 at T421/S424 is coupled with the phosphorylation of ERK in both humans and mice.

We next generated cardiomyocyte-specific Rps6kb1 conditional knockout (cKO) mice by crossing Rps6kb1^fl/fl^ mice (19, 20) with αMHC-Cre mice. We then expressed the ERK2-MEK1 fusion protein at a comparable level in heart tissues between control and cKO mice (Supplemental Figure 3D). Active ERK2 significantly increased the ratios of heart weight to tibia length (HW/TL) and heart weight to body weight (HW/BW) in control mice (Figure 4, E and F). However, Rps6kb1 deficiency in cardiomyocytes dampened this effect. We further examined the histology of hearts from control and cKO mice. The thickness of the left ventricle from cKO mice appears smaller than that from control mice after ERK2-MEK1 overexpression (Figure 4G). Wheat germ agglutinin (WGA) staining showed that Rps6kb1 deficiency remarkably attenuated ERK2-induced enlargement of cardiomyocytes (Figure 4H). A previous report showed that MEK1/ERK pathway activation–induced cardiac hypertrophy led to deceased left ventricle chamber dimension and increased fractional shortening (21). We therefore conducted echocardiography for age-matched mice with or without ERK2-MEK1 overexpression for 2 months (Figure 4I). Consistent with the repressed cardiac hypertrophy, cKO mice with ERK2-MEK1 overexpression displayed normal thickness of the interventricular septum (IVS) and left ventricular posterior wall (LVPW) (Figure 4J), as well as left ventricular internal diameter (LVID) and left ventricular (LV) volume (Figure 4K). Collectively, these results suggest that Rps6kb1 plays an essential role in ERK-induced cardiac hypertrophy in vivo.

### Rps6kb1 is required for pressure overload–induced cardiac hypertrophy in vivo.

**Figure 5:**
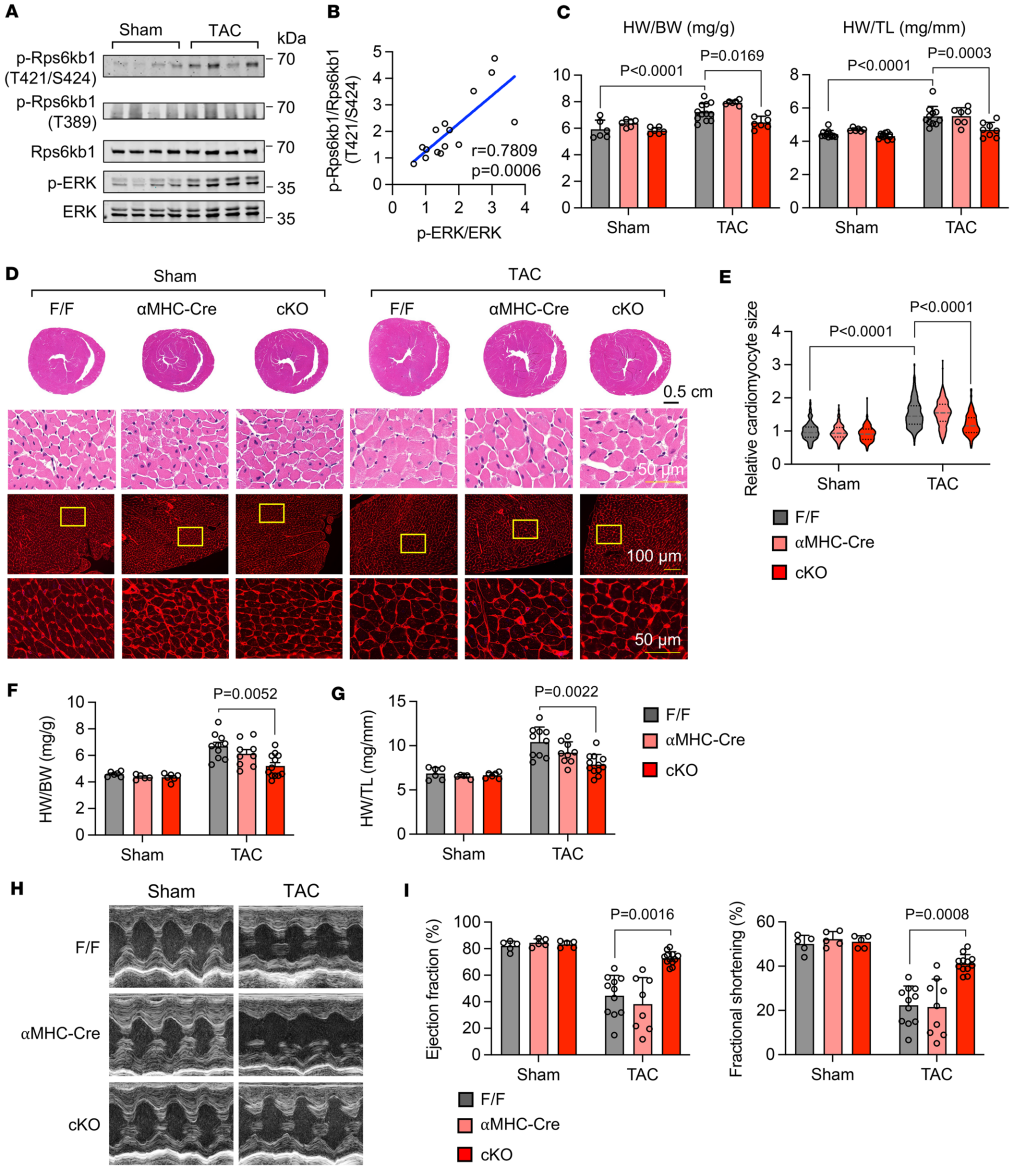
Rps6kb1 is required for pressure overload–induced cardiac hypertrophy. (**A**) Western blotting analysis for heart tissues from mice subjected to either sham or transverse aortic constriction (TAC) surgery for 4 days. (**B**) Correlation analysis between the levels of phosphorylation of Rps6kb1 (T421/S424) and ERK, quantified from **A**. Each dot represents 1 mouse. Both the p-Rps6kb1/Rps6kb1 and p-ERK/ERK ratios were calculated and plotted. Sham, *n* = 3; TAC, *n* = 12. (**C**) Rps6kb1 cKO reduced heart growth in response to pressure overload, as revealed by decreases in the HW/BW and HW/TL ratios. Rps6kb1^fl/fl^, αMHC-Cre, and cKO mice were subjected to either sham or TAC operations for 4 days. *n* = 6–12. (**D**) Representative histological images for heart sections from Rps6kb1^fl/fl^, αMHC-Cre, and cKO mice subjected to either sham or TAC operations for 4 days. Cardiac tissue sections were harvested for H&E (upper) and wheat germ agglutinin (WGA, bottom) staining. Scale bars: 50 μm (top and bottom rows) and 100 μm (middle row). (**E**) Cardiomyocyte cross-sectional area was decreased in Rps6kb1-cKO hearts compared with Rps6kb1^fl/fl^ or αMHC-Cre controls. A total of 150–200 cardiomyocytes for each group was quantified from WGA staining in **D**. (**F**) Rps6kb1 cKO attenuated cardiac hypertrophy in response to pressure overload, as revealed by a reduction in the HW/BW ratio. Rps6kb1^fl/fl^, αMHC-Cre, and cKO mice were subjected to either sham or TAC operations for 4 weeks. *n* = 5–10. (**G**) HW/TL was decreased in Rps6kb1-cKO mice after TAC for 4 weeks. *n* = 5–10. (**H**) Representative cardiac echocardiographic images. Note that the mice were subjected to sham or TAC surgery for 4 weeks. (**I**) Rps6kb1 cKO improved cardiac dysfunction after TAC. Both ejection fraction and fractional shortening were increased in the cKO mice. *n* = 5–11. Pearson’s correlation analysis was conducted for **B**. Two-way ANOVA was conducted, followed by Tukey’s multiple-comparison test for **C**, **E**–**G**, and **I**. Data are presented as mean ± SEM.

Transverse aortic constriction (TAC) is a widely used surgical approach to model cardiac hypertrophic growth in mice (22). Next, we sought to investigate whether Rps6kb1 plays a role in pressure overload–induced cardiac hypertrophy. We first evaluated whether the ERK/Rps6kb1 axis is activated in the heart after TAC. We found that phosphorylation of Rps6kb1 at T421/S424 was increased by TAC (Figure 5A). However, we did not observe any detectable change in mTOR-dependent phosphorylation of Rps6kb1 at T389 in the early stage of cardiac hypertrophy (Figure 5A). Importantly, there was a strong linear correlation between the phosphorylation of Rps6kb1 (T421/S424) and ERK1/2 (Figure 5B), indicating that the activities of ERK and Rps6kb1 are tightly coupled in the heart in response to hemodynamic stress. We then subjected the Rps6kb1-cKO mice along with controls to TAC. HW/BW and HW/TL were strongly repressed in cKO mice after TAC for 4 days (Figure 5C). And the thickness of the ventricular wall from cKO was less compared with the one from controls after TAC (Figure 5D). Accordingly, WGA staining showed that the cross-sectional area of cardiomyocytes in cKO hearts was significantly lower than that of controls after TAC (Figure 5, D and E). Long-term cardiac hypertrophy may lead to heart failure. Next, we examined cardiac hypertrophy and heart function of these mice 4 weeks after TAC. Cardiac hypertrophy at this stage, assessed by HW/BW and HW/TL, was attenuated in the cKO mice (Figure 5, F and G). We found that Rps6kb1-cKO mice manifested improved ejection fraction and fractional shortening after TAC (Figure 5, H and I). Moreover, cardiomyocyte cross-sectional area was reduced in Rps6kb1-cKO mice (Supplemental Figure 4, A and B). At the molecular level, the expression of cardiac hypertrophy makers was reduced (Supplemental Figure 4, C and D), along with decreases in the phosphorylation of Rps6kb1 targets (Supplemental Figure 4, C and E). Taken together, these results indicate that pressure overload–induced cardiac hypertrophy and Rps6kb1 activation rely on MEK/ERK signaling, not mTORC1 activity, and Rps6kb1 is required for the pathological growth of cardiomyocytes under pressure overload.

### ERK directly phosphorylates Rps6kb1 at both the kinase domain and CTD.

**Figure 6:**
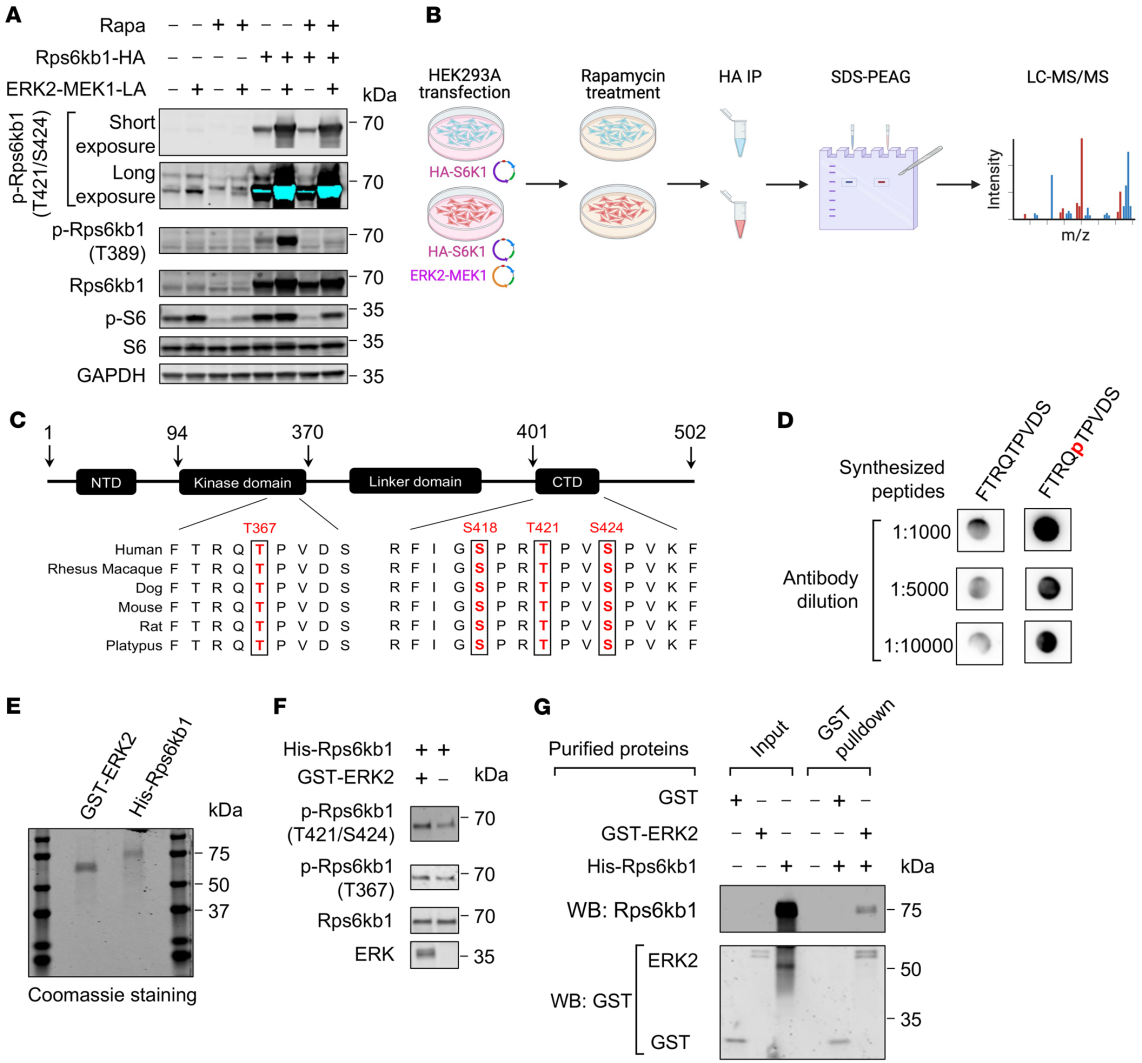
ERK directly phosphorylates Rps6kb1 at T367 and S418/T421/S424. (**A**) Overexpression of activated ERK2 increased the phosphorylation of Rps6kb1. HEK293A cells were transfected with plasmids for myc-ERK2-MEK1 and HA-Rps6kb1 and treated with rapamycin (Rapa). (**B**) Schematic diagram for the experiment to identify the sites of Rps6kb1 phosphorylated by ERK. (**C**) Four phosphorylation sites of Rps6kb1 by ERK were identified in **B**. (**D**) Validation of the newly generated polyclonal antibodies recognizing phosphorylated Rps6kb1 (T367). A short peptide around the T367 residue with or without phosphorylation was synthesized. Dot plot assay showed that the new antibodies had stronger reactivity for the phosphorylated peptides. (**E**) Coomassie blue staining for purified recombinant GST-ERK2 and His-Rps6kb1 proteins. (**F**) Kinase assay showed ERK2 phosphorylated Rps6kb1 at the T367 and S418/T421/S424 sites. Purified His-Rps6kb1 was incubated with GST-ERK2. Phosphorylation of Rps6kb1 was examined by antibodies recognizing the phosphorylated T367 and T421/S424 residues, respectively. (**G**) GST pulldown assay showed the direct interaction between recombinant ERK2 and Rps6kb1.

Based on the observation that phosphorylation of Rps6kb1 and ERK is highly correlated in vitro and in vivo, we hypothesized that ERK may directly phosphorylate Rps6kb1. To test this hypothesis, we first set up to identify potential direct phosphorylation sites of Rps6kb1 by ERK. We overexpressed HA-Rps6kb1 and ERK2-MEK1 fusion protein in HEK293A cells. ERK2-MEK1 overexpression increased the phosphorylation of Rps6kb1 at T421/S424 even under the condition of mTOR inhibition by rapamycin (Figure 6A), which was consistent with the observation in NRVMs. We then immunoprecipitated HA-tagged Rps6kb1 from HEK293A cells with rapamycin treatment and identified protein modifications of Rps6kb1 by LC-MS/MS (Figure 6B). We found 4 phosphorylation modifications of Rps6kb1 that were induced by ERK (Figure 6C and Supplemental Figure 5, A and B). Consistent with the results using commercial antibodies, the T421/S424 sites located in the CTD of Rps6kb1 were phosphorylated. In addition, we showed that phosphorylation of S418 was also governed by ERK. More importantly, we demonstrated that phosphorylation of T367, a site in the kinase domain of Rps6kb1, depended on ERK (Figure 6C and Supplemental Figure 5, A and B).

To explore the role of T367 phosphorylation, we generated polyclonal antibodies that can recognize this phosphorylation site. We synthesized a short stretch of peptides around the T367 residue with or without phosphorylation as antigens (Supplemental Figure 6, A–D). Dot plot assay showed that the antibodies had stronger reactivity for the phosphorylated peptides comparing with the unphosphorylated ones (Figure 6D). We then performed a kinase assay with purified recombinant ERK2 and Rps6kb1. Phosphorylation of T367 and T421/S424 was increased when Rps6kb1 recombinant protein was incubated with ERK2 (Figure 6, E and F). Further GST pulldown assay showed that there was a direct interaction between purified recombinant Rps6kb1 and ERK2 proteins (Figure 6G). Collectively, these results demonstrate that, independent from the activity of mTORC1, ERK2 can directly phosphorylate Rps6kb1 at both T367 in the kinase domain and S418/T421/S424 in the CTD.

### Phosphorylation of T367 and S418/T421/S424 is essential for the full activation of Rps6kb1.

**Figure 7:**
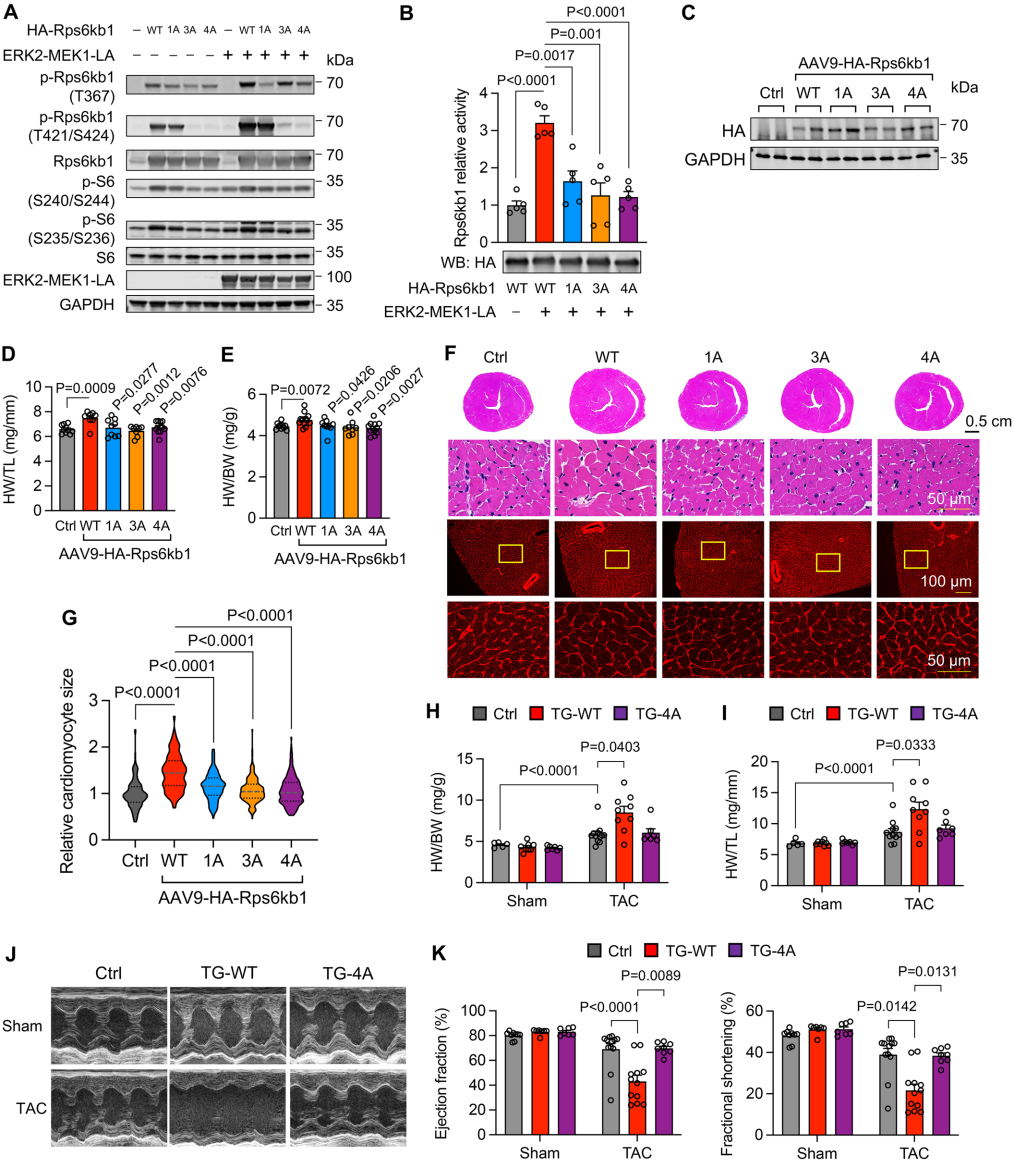
Phosphorylation of T367 and S418/T421/S424 is required for the full activation of Rps6kb1. (**A**) Plasmids expressing WT, T367A (1A), S418A/T421A/S424A (3A), or T367A/S418A/T421A/S424A (4A) Rps6kb1 were transfected into HEK293A cells with or without the expression of ERK2. Western blotting was conducted to evaluate the levels of phosphorylation of Rps6kb1 (T367), Rps6kb1 (T421/S424), and S6. (**B**) Rps6kb1 activity assay showed that 1A, 3A, or 4A mutation impaired its activity. Plasmids expressing WT, 1A, 3A, or 4A Rps6kb1 were transfected into HEK293A cells with or without the expression of ERK2. Rps6kb1 was then pulled down by anti-HA antibody for Rps6kb1 activity assay. (**C**) Validation of the expression of WT and mutated Rps6kb1, respectively, in mouse heart tissues. (**D**) WT, but not mutant, Rps6kb1 overexpression increased the growth of the heart, as revealed by the heart weight/tibia length (HW/TL) ratio. *n* = 8–11. Comparison was conducted between WT and other individual groups. (**E**) The heart weight/body weight (HW/BW) ratio was increased in mice overexpressing WT but not mutant Rps6kb1. *n* = 8–13. Comparison was conducted between WT and other individual groups. (**F**) Representative histological images for heart sections from mice with WT or mutant Rps6kb1 overexpression for 4 months. Cardiac tissues were harvested for H&E (upper) and wheat germ agglutinin (WGA, bottom) staining. Scale bars: 50 μm (top and bottom rows) and 100 μm (middle row). (**G**) Cardiomyocyte cross-sectional area was increased in mice overexpressing WT but not mutant Rps6kb1. A total of 170–192 cardiomyocytes was quantified for each group from WGA staining in **F**. (**H**) Transgenic mice overexpressing either WT (TG-WT) or 4A mutant Rps6kb1 (TG-4A), along with controls, were subjected to sham or TAC for 4 weeks. The HW/BW ratio was significantly increased in TG-WT mice compared with TG-4A mice. *n* = 5–12. (**I**) The HW/TL ratio was elevated in TG-WT mice compared with TG-4A after TAC. *n* = 5–11. (**J**) Representative cardiac echocardiographic images. (**K**) Overexpression of WT Rps6kb1 in the heart aggravated cardiac dysfunction in response to pressure overload, as revealed by decreased in ejection fraction and fractional shortening. *n* = 7–12. Unpaired, 2-tailed Student’s *t* test was conducted for **B**, **D**, **E**, and **G**. Two-way ANOVA was conducted, followed by Tukey’s multiple-comparison test for **H**, **I**, and **K**. Data are presented as mean ± SEM.

We next asked whether the phosphorylation of T367 and S418/T421/S424 is required for the activation of Rps6kb1. To address this question, we introduced mutations to the Rps6kb1 expression plasmid to abolish ERK-dependent phosphorylation of Rps6kb1 (Supplemental Figure 7), including T367A (1A), S418A/T421A/S424A (3A), and T367A/S418A/T421A/S424A (4A). These plasmids were cotransfected with the ERK2-MEK1 fusion protein plasmid into HEK293A cells. As expected, phosphorylation of T367 was decreased in both 1A and 4A mutants, and phosphorylation of T421/S421 was reduced in both 3A and 4A mutants (Figure 7A). Importantly, we found that phosphorylation of S6, a target of Rps6kb1, was repressed in cells with all types of mutated Rps6kb1 (Figure 7A), indicating impairment of Rps6kb1 activity. Next, to directly assess the activity of Rps6kb1, we purified WT and mutant Rps6kb1 proteins from HEK293A cells and incubated them with Rps6kb1 target in vitro. We found that the 1A, 3A, or 4A mutation almost entirely disrupted ERK-induced activation of Rps6kb1 (Figure 7B).

We further evaluated the role of WT and mutant Rps6kb1 in cardiomyocytes in vivo. WT, 1A, 3A, or 4A mutant Rps6kb1 was introduced into cardiomyocytes by AAV9 infection in neonatal mice (Figure 7C). We found that WT Rps6kb1 further increased the growth of cardiomyocytes after overexpression for 4 months, manifested by elevated HW/TL and HW/BW ratios (Figure 7, D and E). In contrast, the 1A, 3A, or 4A mutation had no effect (Figure 7, D and E). Accordingly, the cross-sectional area of cardiomyocytes was significantly increased in the heart with WT Rps6kb1 overexpression, but not with the 1A, 3A, or 4A mutation (Figure 7, F and G).

To explore the function of ERK-mediated phosphorylation in Rps6kb1 in pathological cardiac hypertrophy, we generated cardiomyocyte-specific WT and 4A mutant Rps6kb1–overexpressing mice, respectively, using the doxycycline-inducible system (Supplemental Figure 8, A and B) (23). We found that WT, but not 4A mutant, Rps6kb1 overexpression for a short term further augmented TAC-indued cardiac hypertrophy, as revealed by increases in HW/BW and HW/TL (Figure 7, H and I). We next performed echocardiography to assess heart function. We found that WT Rps6kb1 overexpression impaired cardiac dysfunction (Figure 7, J and K). Moreover, cardiomyocyte cross-sectional area was increased in WT Rps6kb1–overexpressing mice (Supplemental Figure 8, C and D). Accordingly, the expression levels of Acta1, βMHC, ANP, and Rcan1.4 were further augmented after TAC in WT Rps6kb1–overexpressing mice, compared with control and 4A overexpression (Supplemental Figure 9, A and B). In addition, the levels of p-eIF4B and p-S6 were further increased upon WT Rps6kb1 overexpression (Supplemental Figure 9, A and C), indicating higher Rps6kb1 activity in these mice. Collectively, these findings suggest that ERK-dependent phosphorylation of Rps6kb1 is indispensable for its full activity in vitro and in vivo. Under pathological conditions, Rps6kb1 overactivation in cardiomyocyte may aggregate cardiac hypertrophy and impair heart function.

## Discussion

In this study, we revealed an ERK-dependent signaling pathway that phosphorylates and activates Rps6kb1 during cardiac hypertrophic growth. We showed that the MEK/ERK signaling axis is coupled with the activation of Rps6kb1 both in vitro and in vivo. We further demonstrated that ERK directly interacts and phosphorylates Rps6kb1 at multiple sites, including T367 in the kinase domain and S418/T421/S424 in the CTD. Importantly, phosphorylation of either T367 or S418/T421/S424 is required for the full activation of Rps6kb1. Taken together, these findings revealed a signaling pathway, ERK/Rps6kb1, which plays an essential role in protein synthesis and cardiomyocyte hypertrophic growth under hemodynamic stress (Supplemental Figure 10).

In contrast with our findings, a previous study reported that Rps6kb1 had no effect on TAC-induced cardiac hypertrophy (24). This discrepancy may be caused by different mouse models employed. McMullen et al. used a whole-body germline knockout mouse model and conducted TAC. Compelling evidence has shown that various cells, including fibroblasts (25), immune cells (26), and endothelial cells (27), play important roles in cardiac hypertrophic growth and heart failure. In addition, Rps6kb1 whole-body knockout mice manifest abnormal gain of body weight (28). Previous studies showed that Rps6kb1 deletion had profound effects on the function of the liver (29), skeletal muscle (30), and neurons (31). All the above effects may affect hypertrophic response in the heart in the TAC model when a whole-body Rps6kb1 deletion model is used. In our study, we generated cardiomyocyte-specific Rps6kb1-cKO mice, which can largely exclude the effect of other types of cells and organs. Moreover, we subjected these mice to 2 models of cardiac hypertrophic growth and demonstrated that Rps6kb1 in cardiomyocytes plays an indispensable role in cardiac hypertrophy.

TAC-induced cardiac hypertrophy is broadly used to induce cardiac remodeling and heart failure (22). Interestingly, in this model we did not find the induction of T389 phosphorylation of Rps6kb1, indicating that mTORC1 signaling is not further activated but is vital to maintain cardiac homeostasis (11). On the contrary, ERK-driven phosphorylation of Rps6kb1 is increased, indicating that ERK is responsible for Rps6kb1 activation in TAC-induced cardiac hypertrophic growth. Under cardiomyopathy, ERK signaling may be stimulated by neurohumoral factors, GPCRs, and cytoskeletal reorganization (5, 32). These findings may shift our conventional understanding of Rps6kb1 activation from mTORC1 to ERK and may be instructive in developing Rps6kb1-based treatment for hypertensive heart disease.

In response to extracellular cues, 2 main signaling pathways, Ras/ERK and PI3K/mTOR, are activated to control cell survival, differentiation, proliferation, metabolism, and motility (33). Previous reports demonstrated that the cross-activation between these 2 pathways relies on ERK- or RSK-mediated phosphorylation of TSC2 (34), which ultimately activates mTORC1 to phosphorylate Rps6kb1 and 4EBP1. Moreover, RSK may directly phosphorylate S6, independent from Rps6kb1 (16), suggesting complicated crosstalk between ERK and mTOR signaling pathways. However, our study established an mTORC1-independent connection between these 2 pathways, in which ERK directly phosphorylates and activates Rps6kb1. This ERK/Rps6kb1 signaling axis may exist in other eukaryotic cells and have broad implications for various diseases, including cancer and diabetes. Furthermore, under different conditions, the MEK/ERK axis, mTOR, RSK, and Rps6kb1 may be activated to different levels, thereby fine-tuning the activation of downstream targets, like S6 and eIF4B.

Rps6kb1 consists of multiple functional domains, including nuclear localization sequence (NLS, specific for p85-Rps6kb1), N-terminal domain (NTD, containing the mTOR-sensitive TOS-motif), kinase domain, linker domain, and CTD (containing the autoinhibitory motif). The current model for the process of Rps6kb1 activation includes 3 steps. First, multi-phosphorylation of the CTD releases the NTD and kinase domains. Second, mTOR accesses the linker domain to phosphorylate T389. Third, PDK1 further phosphorylates the T229 residue in the kinase domain to maximize the activity of Rps6kb1 (35). Regarding the first step, it has been reported that under stimulation with IFN-γ, CDK5 is responsible for phosphorylating the CTD (36). Another 2 studies suggest that CDK1, PKC, and MAP kinase may be involved, albeit the evidence is vague (37, 38). Here, we demonstrated that ERK is critical for the phosphorylation of the CTD under growth conditions in cardiomyocytes. We also identified a phosphorylation site in the kinase domain of Rps6kb1 (T367) and demonstrated that its phosphorylation is indispensable for ERK-induced activation of Rps6kb1. These findings may change our conventional view regarding the activation of Rps6kb1. Under the condition of modest activation of mTORC1, ERK may confer Rps6kb1 basal activity by phosphorylating T367 in the kinase domain and the S418/T421/S424 sites in the CTD (Supplemental Figure 10). On the other hand, when mTORC1 is fully activated, ERK may prime the activation of Rps6kb1 by first phosphorylating the S418/T421/S424 sites in the CTD. Then, mTORC1 may access Rps6kb1 to phosphorylate T389 (35), and ERK may continue to phosphorylate T367 to fully boost the activity of Rps6kb1. Additional experiments are warranted to test this model of Rps6kb1 activation.

In conclusion, we delineated a signaling pathway that activates Rps6kb1, promoting protein synthesis and cell growth. Since this ERK/Rps6kb1 signaling axis plays a critical role in the process of cardiac hypertrophy and heart failure, innovative therapeutic strategies may be designed to target this pathway to ameliorate pathological cardiac remodeling and heart failure. Furthermore, this mTORC1-independent activation of Rps6kb1 may advance our understanding of the crosstalk between Ras/ERK and PI3K/mTOR signaling pathways and provide insights into protein synthesis and cell growth under both healthy and diseased conditions.

## Methods

Further information can be found in Supplemental Methods.

### Sex as a biological variable.

For rat experiments, both males and females were used to isolate NRVMs. For mouse experiments, only male mice were examined to reduce potential variability in phenotype. It is unknown whether the findings are relevant for female mice.

### Animals.

Mice were bred onto the C57BL/6N background and maintained under a 12-hour light/dark cycle in a mouse facility with temperature control. All mice had free access to water and chow food (Teklad, 2916). For animal surgeries, mice were anesthetized by a cocktail of ketamine (100 mg/kg, intraperitoneal injection) and xylazine (5 mg/kg, intraperitoneal injection) 30 minutes before operation. Mice were gently restrained and deeply anesthetized with pentobarbital (100 mg/kg, intraperitoneal injection) at the termination of experiments. NRVMs were isolated from 1- to 2-day-old Sprague-Dawley rats (Charles River Laboratories) as previously reported (39).

### Western blotting.

Total proteins were prepared from cells or cardiac tissues using RIPA lysis and extraction buffer (Thermo Fisher Scientific, 89900), supplemented with protease and phosphatase inhibitors (Thermo Fisher Scientific, 88669). Protein concentration was quantified with a BCA kit (Thermo Fisher Scientific, 23225). Equal total proteins of each sample were loaded onto 26-well Criterion TGX precast gels (Bio-Rad, 4%–20%, 5671095) and transferred onto nitrocellulose membranes (Bio-Rad, 1704157). After blocking with 5% nonfat milk or 3% BSA for 1 hour at room temperature, membranes were incubated with primary antibodies overnight at 4°C, followed by incubation with secondary antibodies for 1 hour and imaging with an Odyssey scanner (Li-Cor). The following antibodies were used: GAPDH (Fitzgerald, 10R-G109A), Rcan1 (Sigma-Aldrich, D6694), ERK1/2 (Cell Signaling Technology, 4696), p-ERK1/2 T202/Y204 (Cell Signaling Technology, 9101), Rps6kb1 (Cell Signaling Technology, 2708), p-Rps6kb1 T389 (Cell Signaling Technology, 9206), p-Rps6kb1 T421/S424 (Cell Signaling Technology, 9204), S6 (Cell Signaling Technology, 2317), p-S6 S240/S244 (Cell Signaling Technology, 5364), p-S6 S235/S236 (Cell Signaling Technology, 4858), 4EBP1 (Cell Signaling Technology, 9644), p-4EBP1 T37/T46 (Cell Signaling Technology, 2855), Myc (Santa Cruz Biotechnology, sc-40), HA (Cell Signaling Technology, 3724), eIF4B (Cell Signaling Technology, 3592), p-eIF4B S422 (Cell Signaling Technology, 3591), p-eIF4B S406 (Cell Signaling Technology, 5399), Acta1 (Sigma-Aldrich, A2066), βMHC (Sigma-Aldrich, M8421), ANP (Proteintech, 27426-1-AP), BNP (Abcam, ab19645), GST (Cell Signaling Technology, 2622), IRDye 800 CW goat anti-rabbit secondary antibody (Li-Cor, 925-32211), and Alexa Fluor 680–conjugated anti-mouse secondary antibody (Thermo Fisher Scientific, A21057). Specific polyclonal antibodies against Rps6kb1 phospho-T367 (synthetic peptide FTRQ[pT]PVDS) were produced by Abclonal.

### ^3^H-leucine incorporation assay.

NRVMs were cultured with serum-free medium in 6-well plates. After 24 hours, Rps6kb1 was silenced by siRNA transfection. L-[3,4,5-^3^H]-leucine (PerkinElmer, NET460A001MC, 2 μCi/mL), Adeno-GFP, and Adeno-caMEK1 were added to the medium. After another 24 hours, cells were washed 3 times with ice-cold PBS and incubated with 2 mL trichloroacetic acid (LabChem, LC262302, 10%) for 30 minutes at 4°C with gentle agitation. After 2 washes with ice-cold 95% ethanol, samples were incubated with 1 mL NaOH (0.5N) at 37°C for 18–24 hours with gentle agitation. Finally, samples were neutralized with 1 mL HCl (0.5N), and all contents were transferred to scintillation vials. After mixing with scintillation solution (MP Biomedicals, EcoLite, 882475, 18 mL), radioactivity was detected by a scintillation counter (Beckman, LS5000TA).

### Statistics.

Data are presented as mean ± SEM. Normality of data distribution was evaluated by using the Shapiro-Wilk test. Two-tailed Student’s *t* test was performed to compare differences between 2 groups. For multiple group comparisons with 1 variable, 1-way ANOVA was conducted, followed by Tukey’s multiple-comparison test. For multiple group comparisons with more than 2 variables, 2-way ANOVA was conducted, followed by Tukey’s multiple-comparison test. A *P* value of less than 0.05 was considered statistically significant. Statistical analyses were performed using GraphPad Prism software 8.4.2.

### Study approval.

All animal procedures conformed to the NIH *Guide for the Care and Use of Laboratory Animals* (National Academies Press, 2011) and were approved by the Institutional Animal Care and Use Committee of the University of Texas Southwestern Medical Center (UTSW) and City of Hope National Medical Center (COH).

### Data availability.

MS data have been deposited to MassIVE (https://massive.ucsd.edu/ProteoSAFe/static/massive.jsp?redirect=auth) under accession numbers MSV000089177, MSV000089181, MSV000089182, and MSV000089184. Values for all data points in graphs are reported in the Supporting Data Values file. The data that support the findings of this study are available from the corresponding author upon reasonable request.

## Author contributions

CL and ZVW conceived and designed the study. CL and PZ performed most experiments with the help from JAC (cloning), WS (kinase activity assay), XL, HZ, XG, and AL (phosphoproteomics analysis), MV, LAS, NR, MH, MIA, and JL (tissue samples), KZ, GZ, QL, YF, and WME (mouse surgery and echocardiography), and ELN and XNS (cell culture). CL and ZVW wrote the manuscript with help from YD, TGG, and PES. All authors approved the manuscript.

## Funding support

This work is the result of NIH funding, in whole or in part, and is subject to the NIH Public Access Policy. Through acceptance of this federal funding, the NIH has been given a right to make the work publicly available in PubMed Central.

- NIH grant R01 HL-171309 (to ZVW).
- NIH grants R01-DK55758, R01-DK099110, and R01-DK127274 (to PES).
- American Heart Association grants 23TPA1077069 and 947398 (to ZVW).
- American Heart Association grant 24TPA12988803 (to YD).
- American Diabetes Association grant 7-20-IBS-218 (to ZVW).
- American Diabetes Association Post-Doctoral Fellowship 7-21-PDF-158 (to CL).
- American Diabetes Association grant 1-19-JDF-082 (to YD).
- VA Meric Award I01BX005625 and I01CX002406 (to JL).

## Supplementary Material

Unedited blot and gel images

Supplemental data

Supplemental tables 1-9

Supporting data values

## Version 1 01/08/2026

In-Press Preview

## Version 2 02/23/2026

Electronic publication
