# Supplementary material for: Phosphoproteomics identification of ERK-dependent activation of Rps6kb1 in cardiac hypertrophy
Source: JCI Insight. 2026 Jan 8;11(4):e190760. doi: 10.1172/jci.insight.190760 (PMC12956007; doi:10.1172/jci.insight.190760)

Full unedited gel for Figure 1C

Protein ladder used in this figure
(ThermoFisher,#26619)

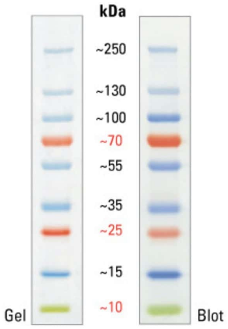

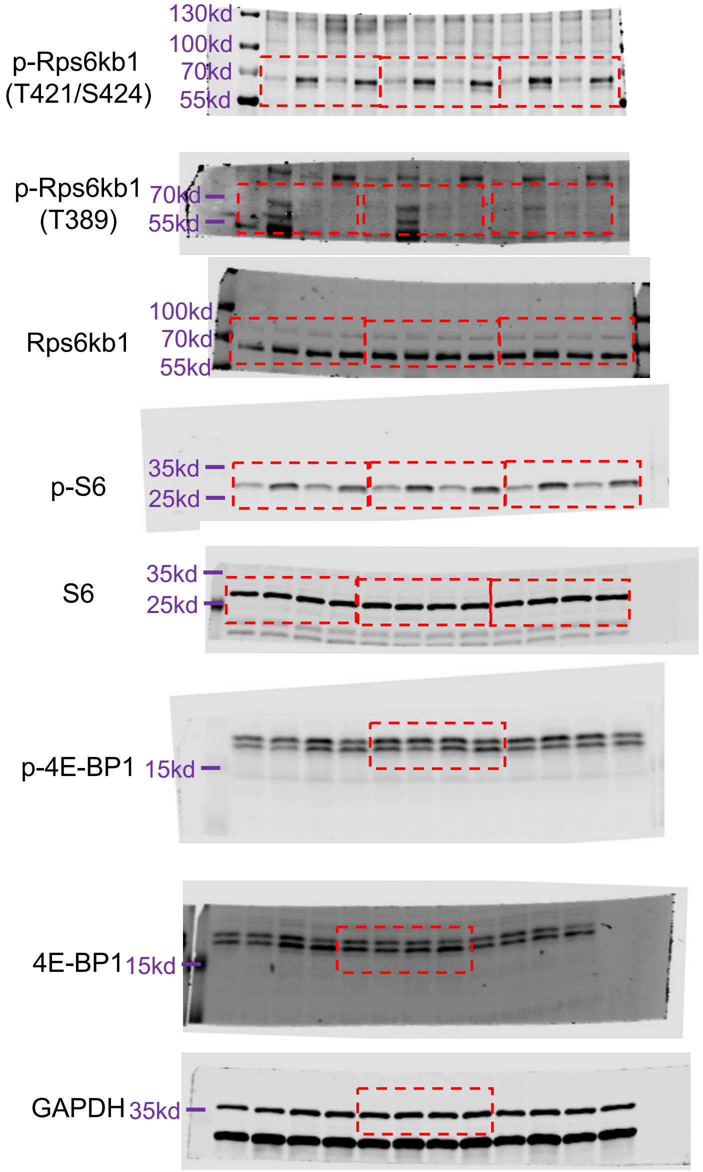

Full unedited gel for Figure 2B

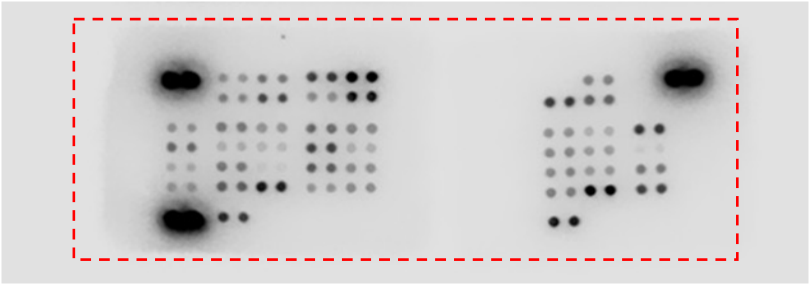

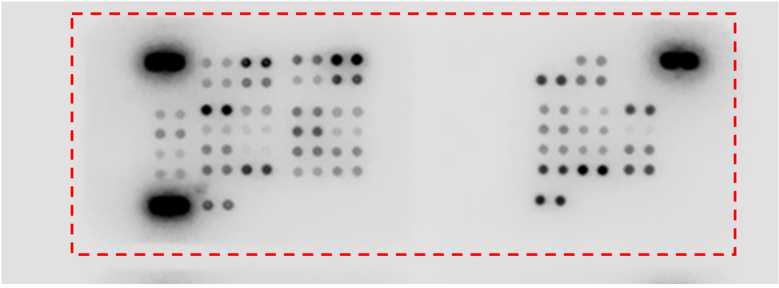

Full unedited gel for Figure 2C

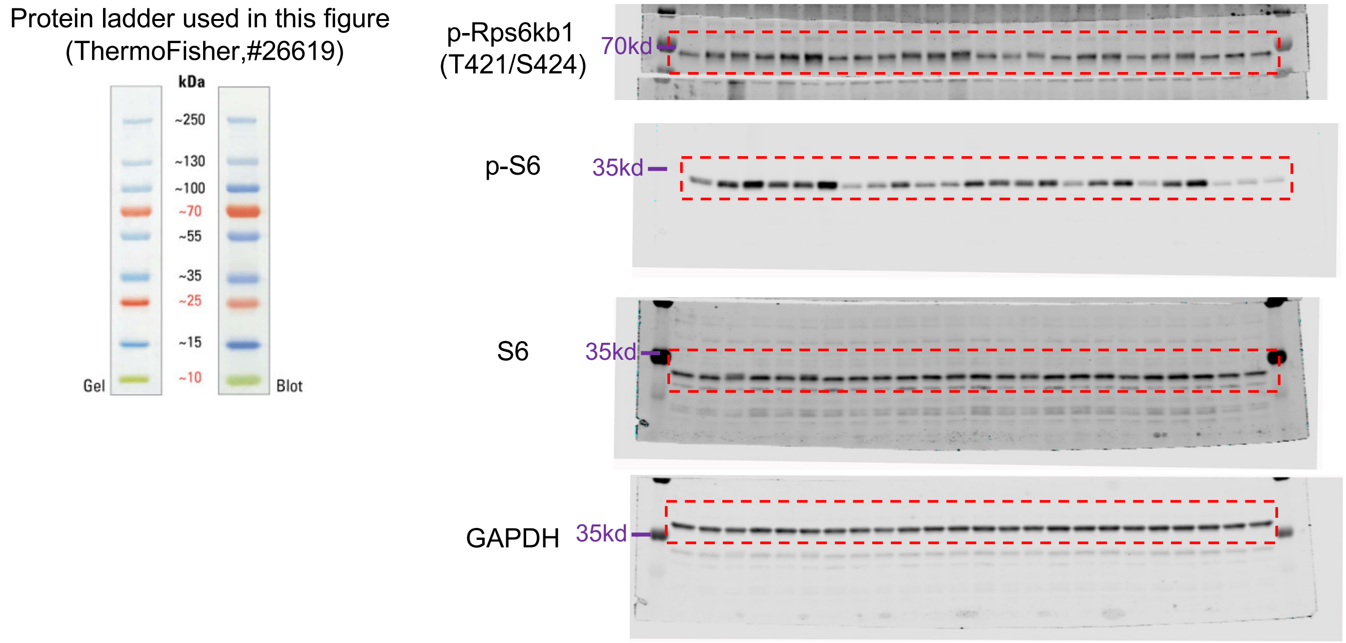

Full unedited gel for Figure 2E

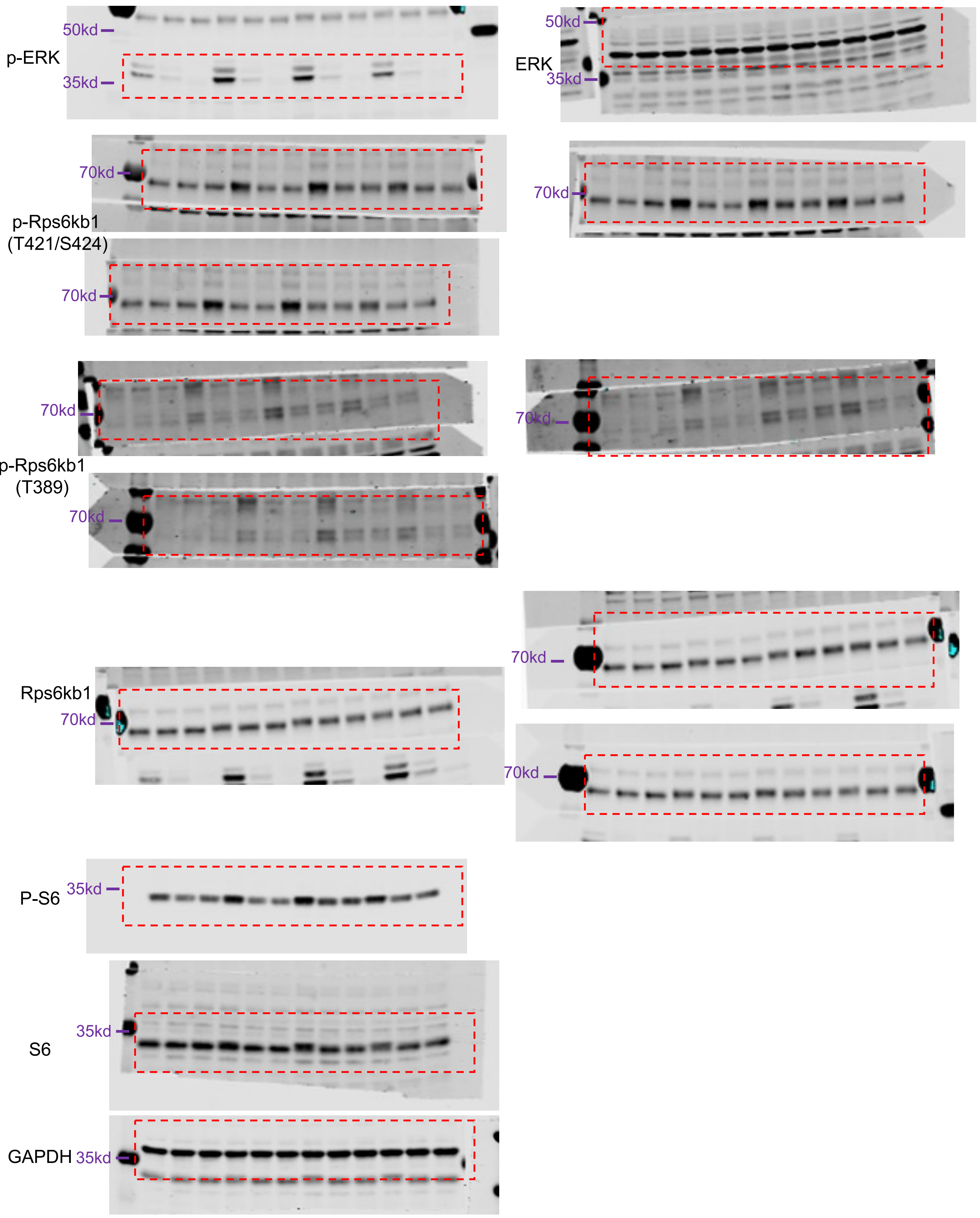

Full unedited gel for Figure 2F

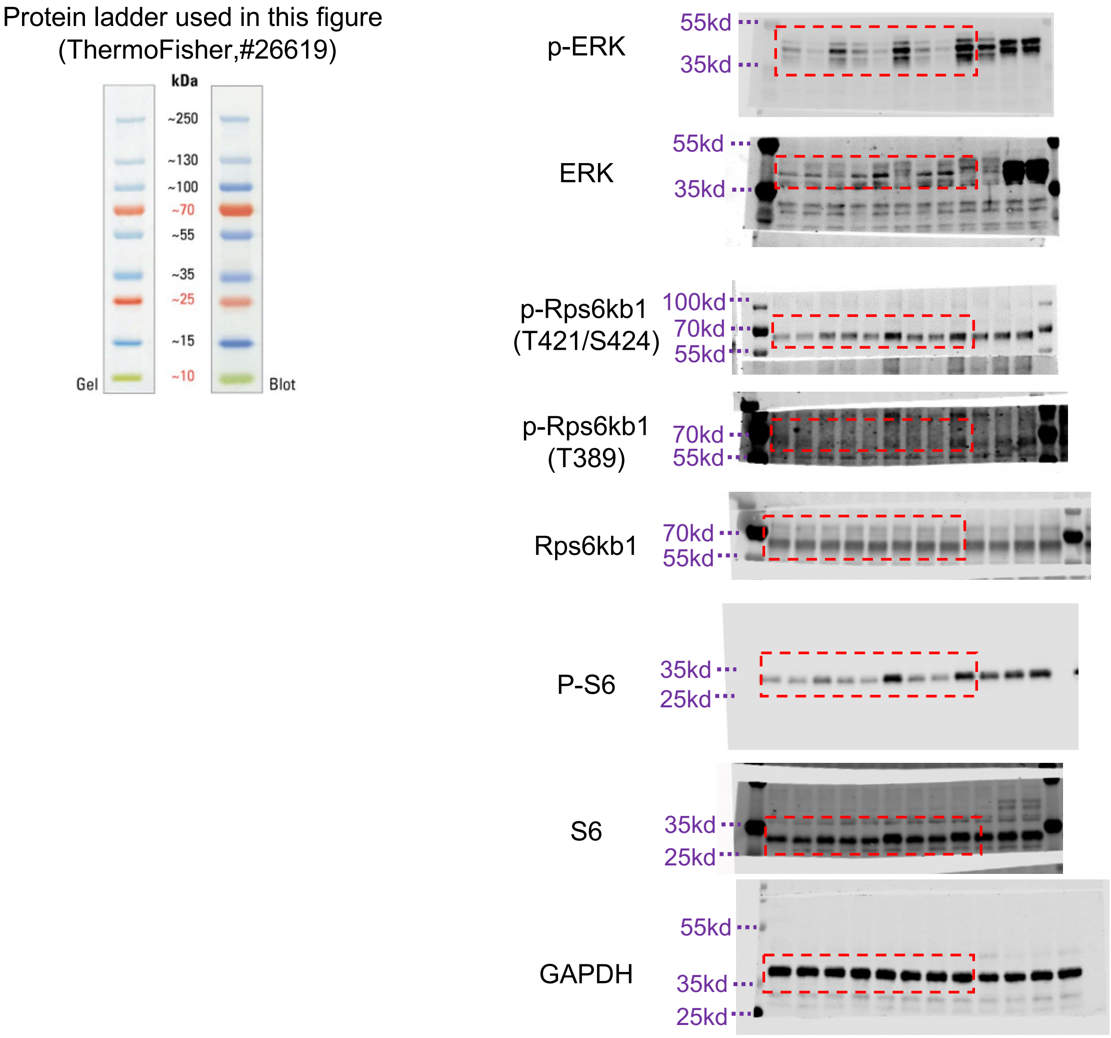

Full unedited gel for Figure 3A

Protein ladder used in this figure
(ThermoFisher,#26619)

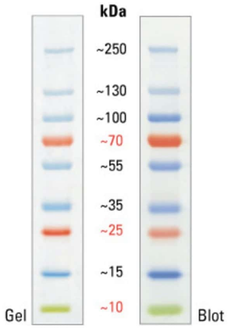

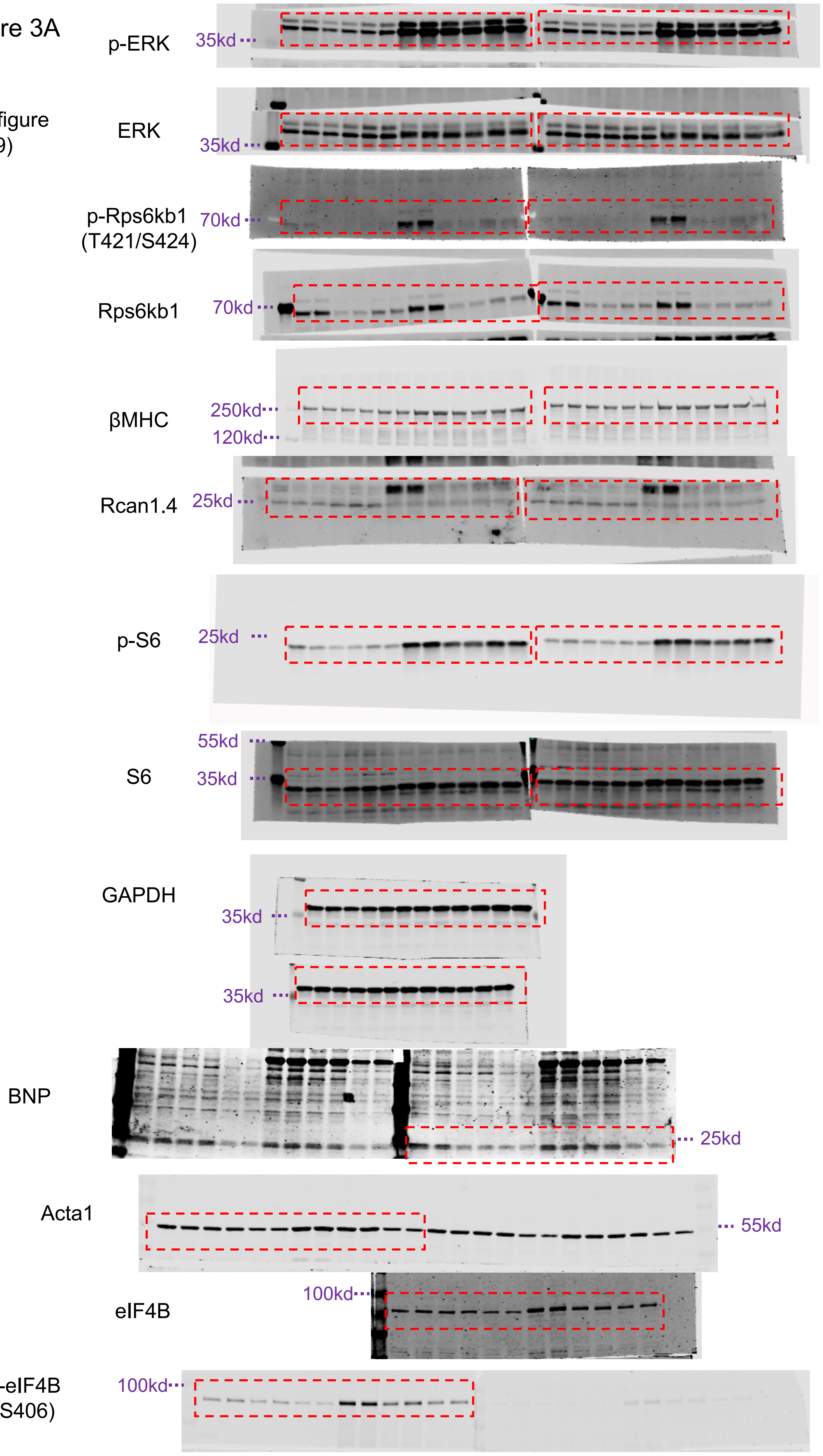

Full unedited gel for Figure 4C

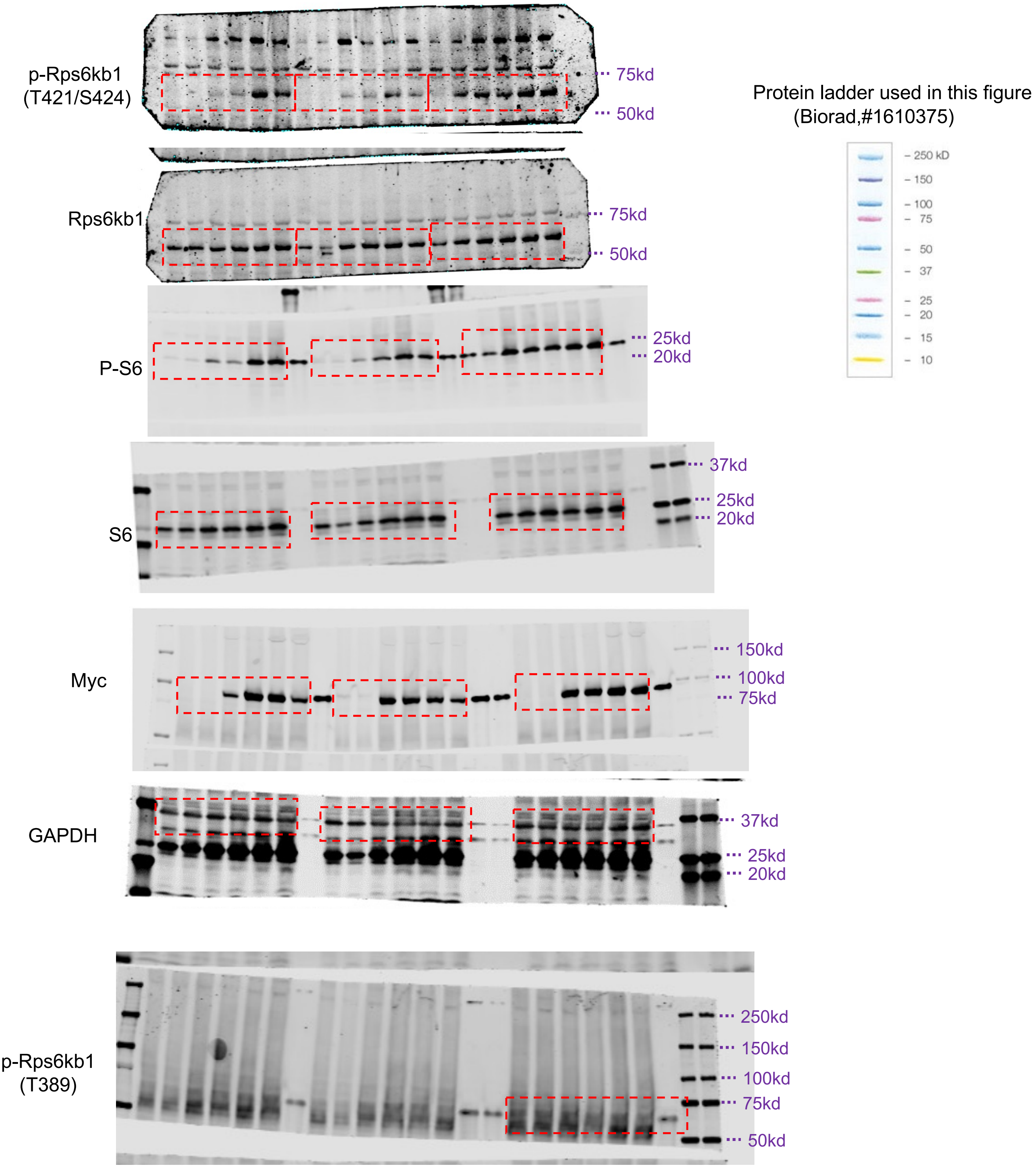

Full unedited gel for Figure 5A

Protein ladder used in this figure
(Biorad,#1610375)

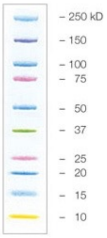

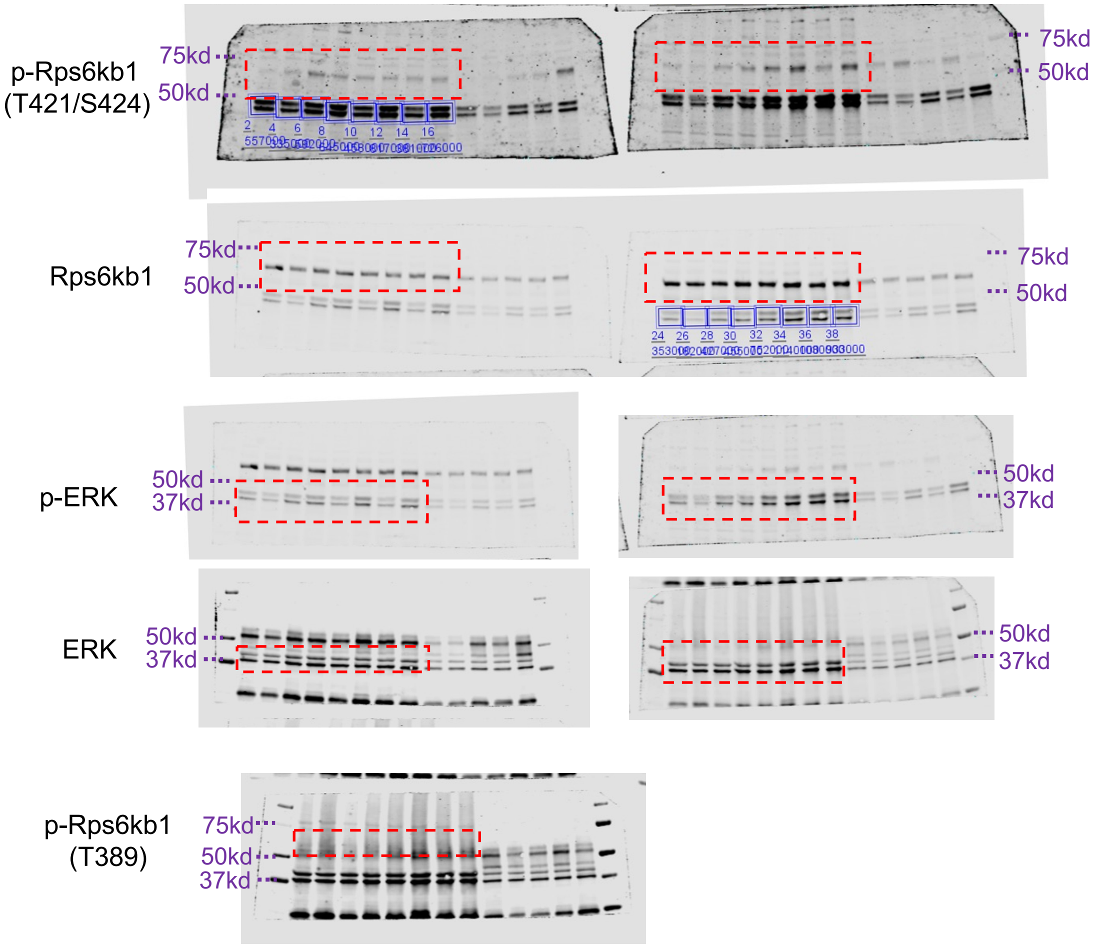

Full unedited gel for Figure 6A

Protein ladder used in this figure
(ThermoFisher,#26619)

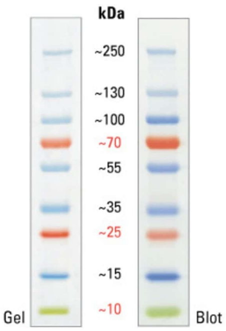

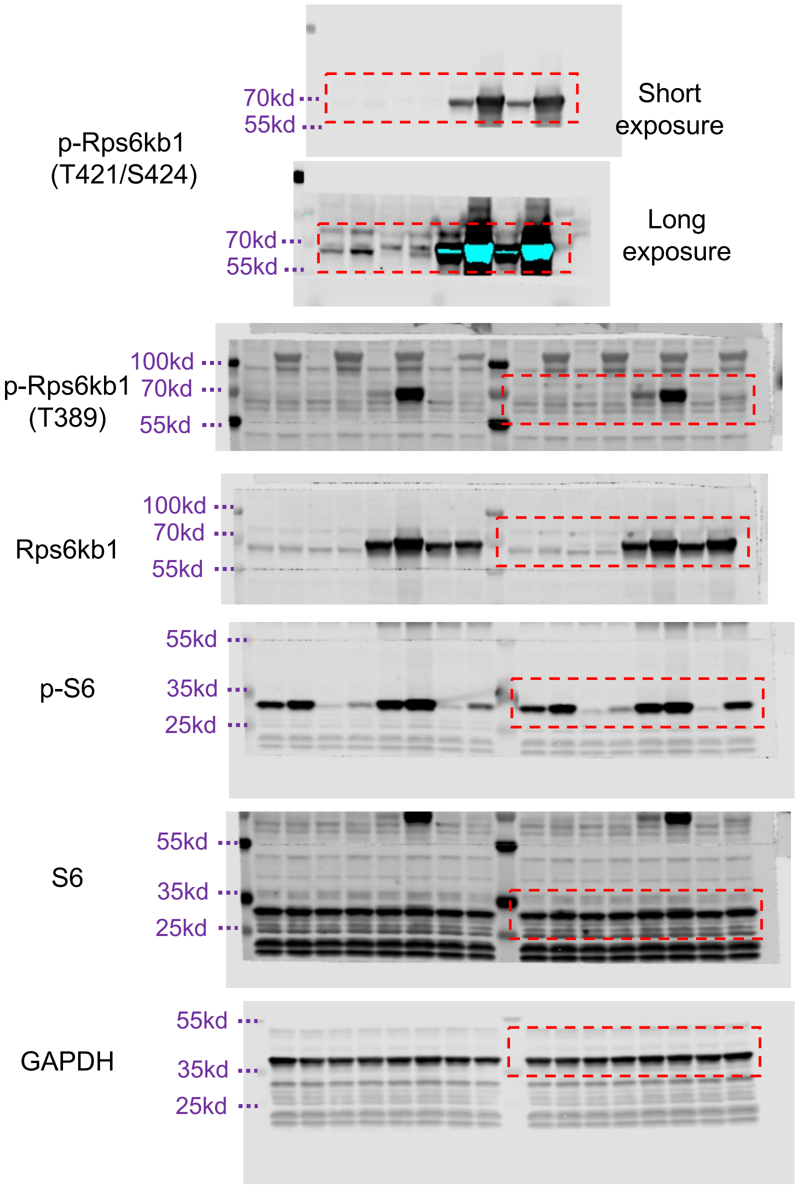

Full unedited gel for Figure 6E

Protein ladder used in this figure
(Biorad,#1610375)

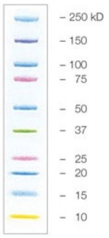

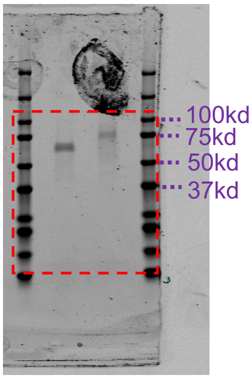

Figure 6F

p-Rps6kb1
(T421/S424)

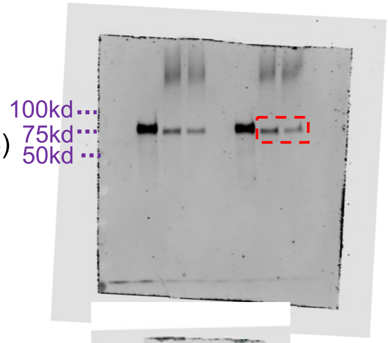

p-Rps6kb1
(T367)

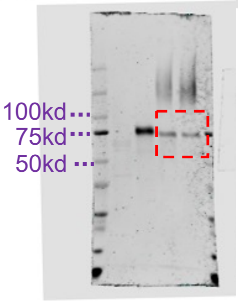

Rps6kb1

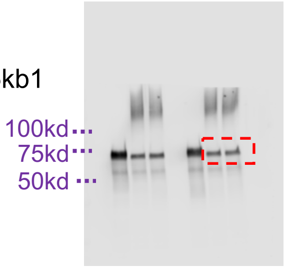

ERK

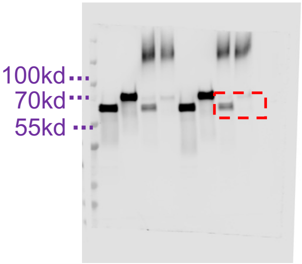

Figure 6G

Rps6kb1

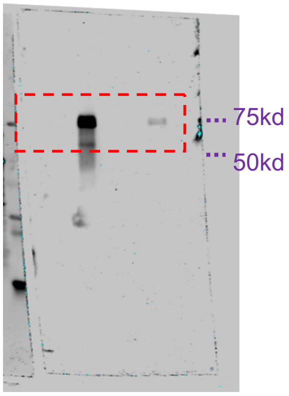

GST

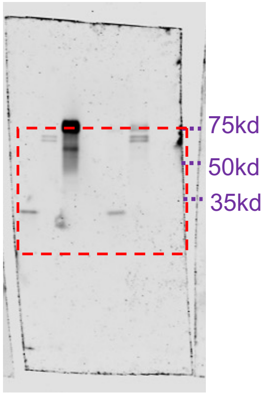

Full unedited gel for Figure 7A

Protein ladder used in this figure (ThermoFisher,#26619)

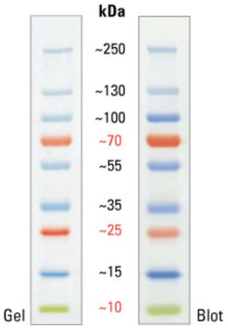

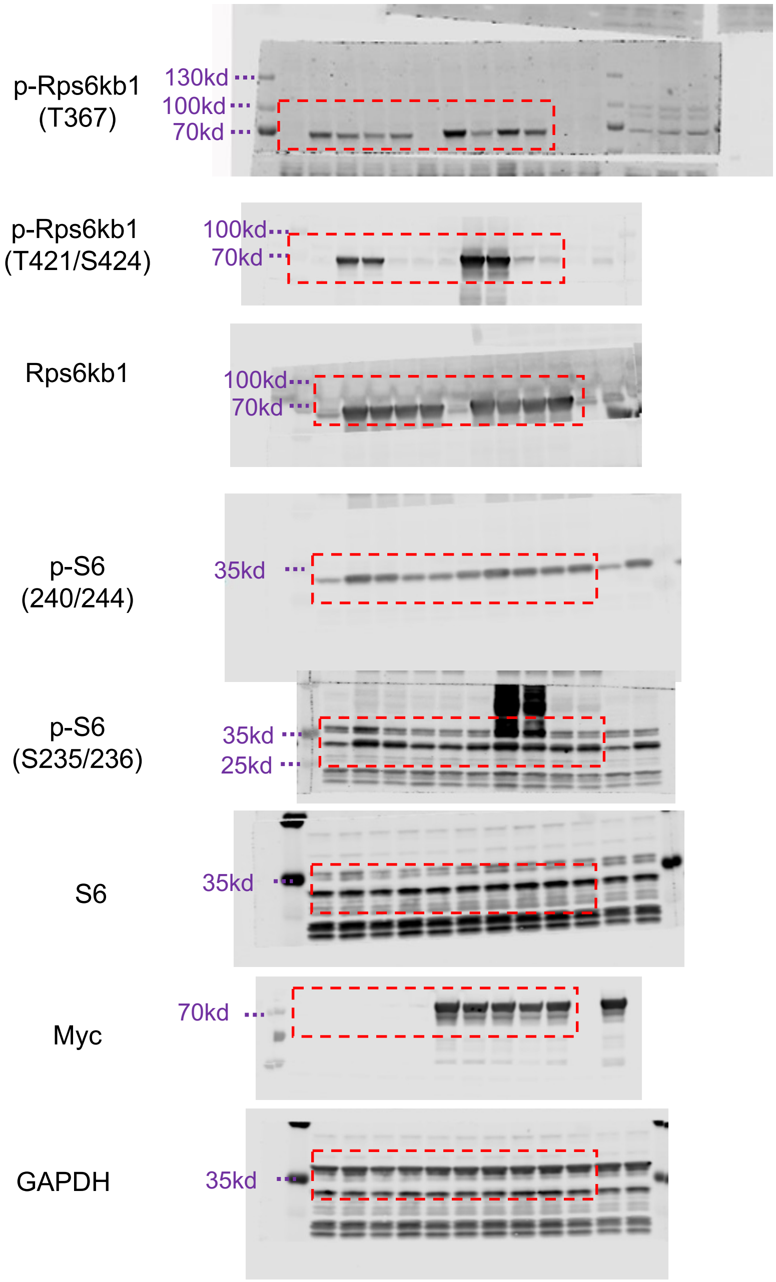

Figure 7B

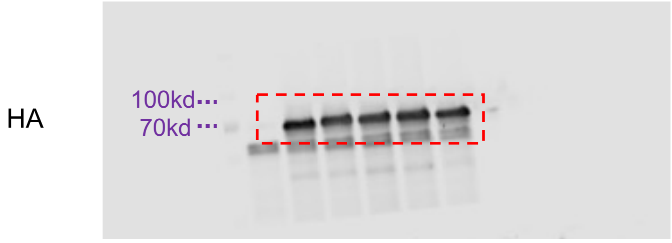

Figure 7C

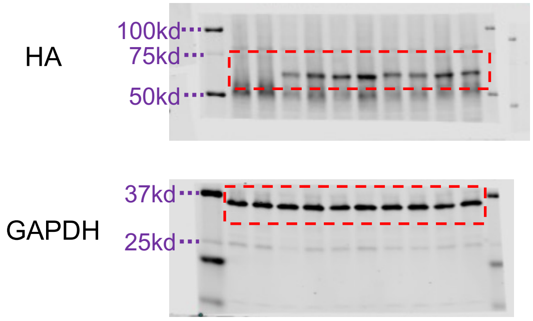

Full unedited gel for Supplemental Figure S2A

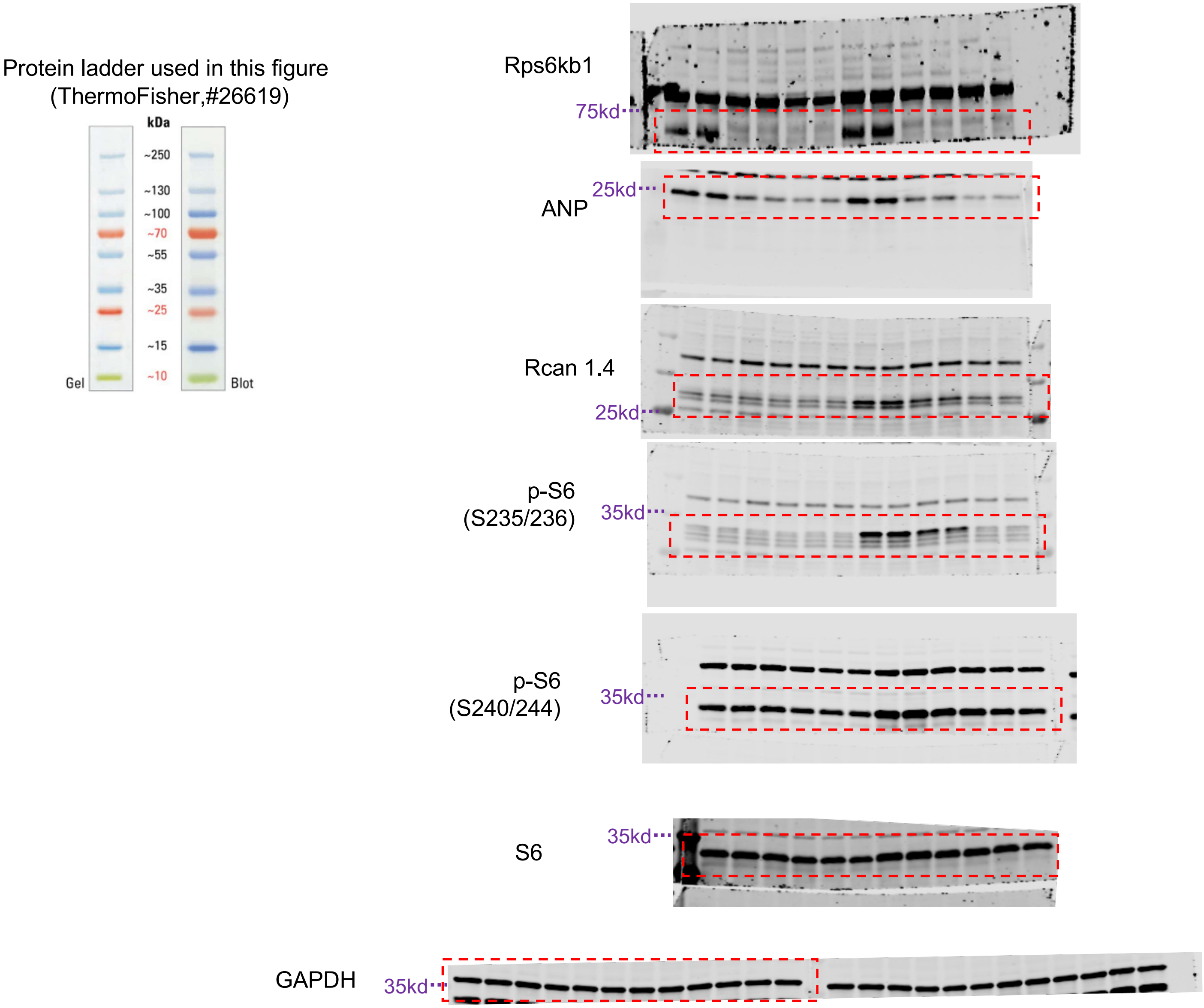

Full unedited gel for Supplementary Figure S3C

Protein ladder used in this figure (ThermoFisher,#26619)

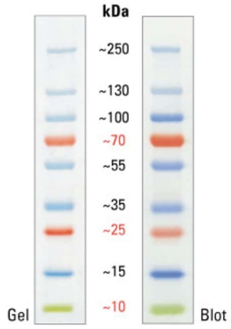

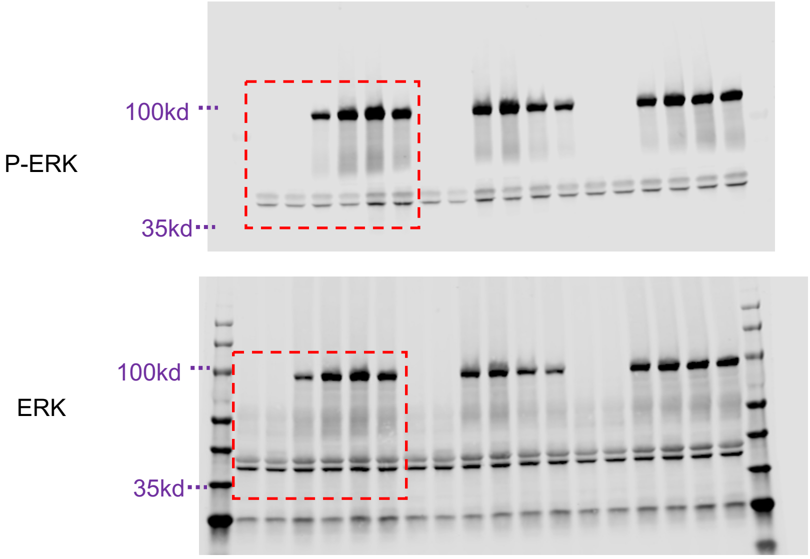

Full unedited gel for Supplemental Figure S3D

Protein ladder used in this figure
(ThermoFisher,#26619)

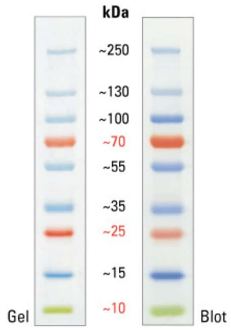

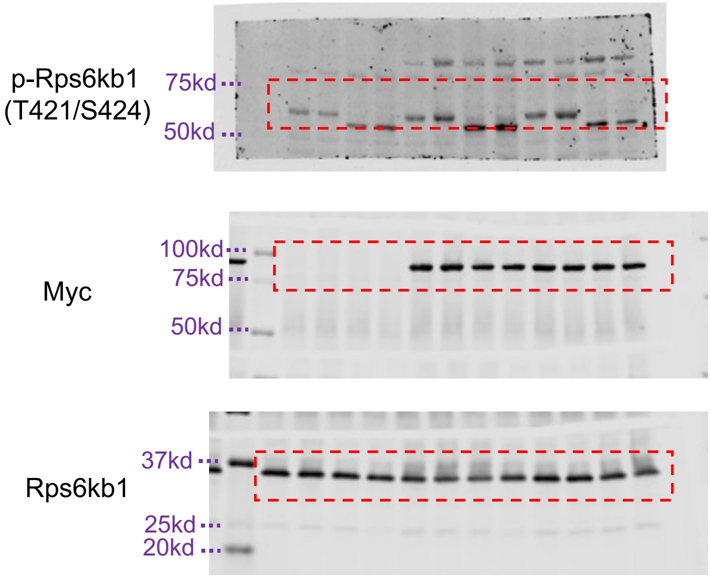

Full unedited gel for Supplemental Figure S4C

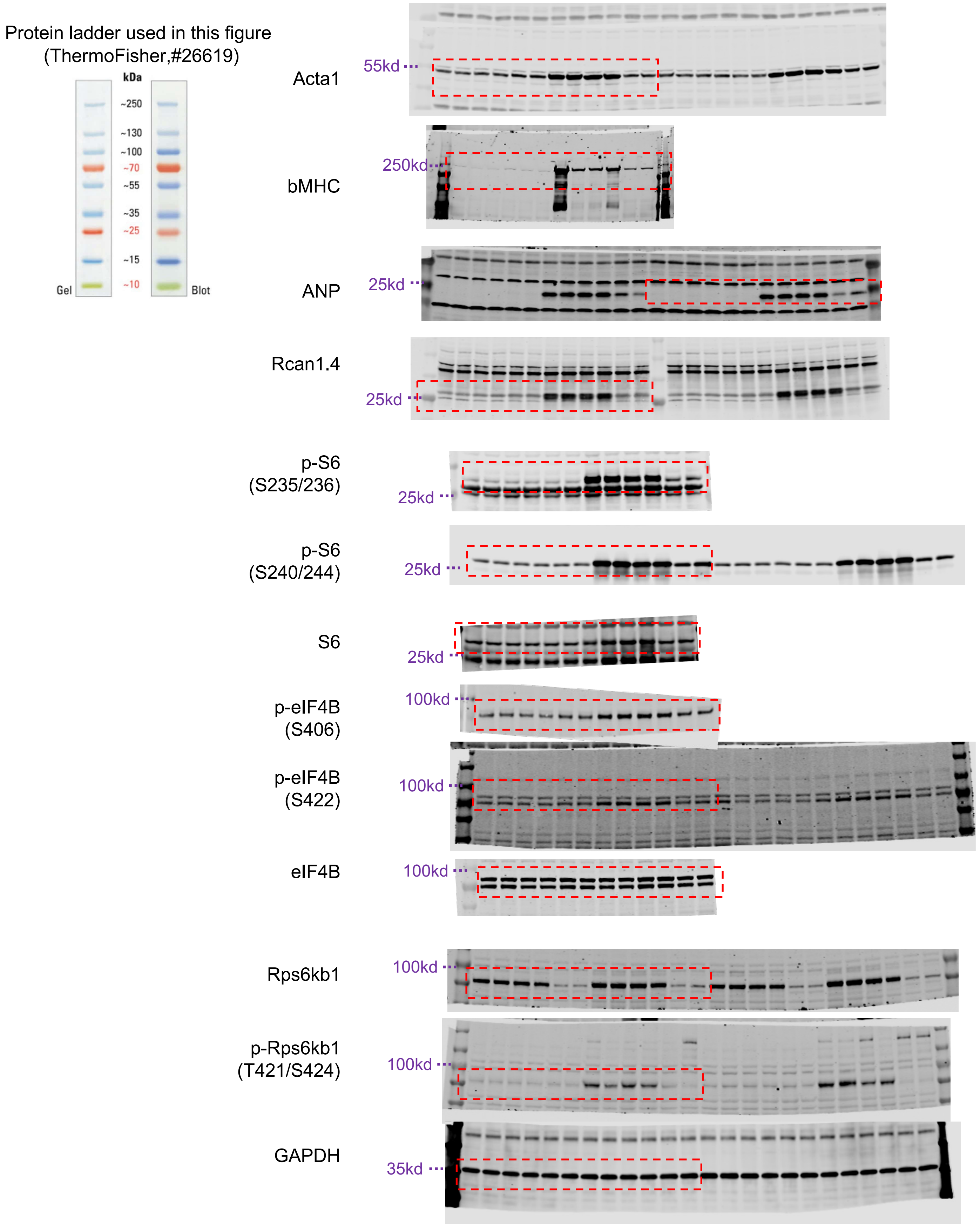

Full unedited gel for Supplemental Figure S9A

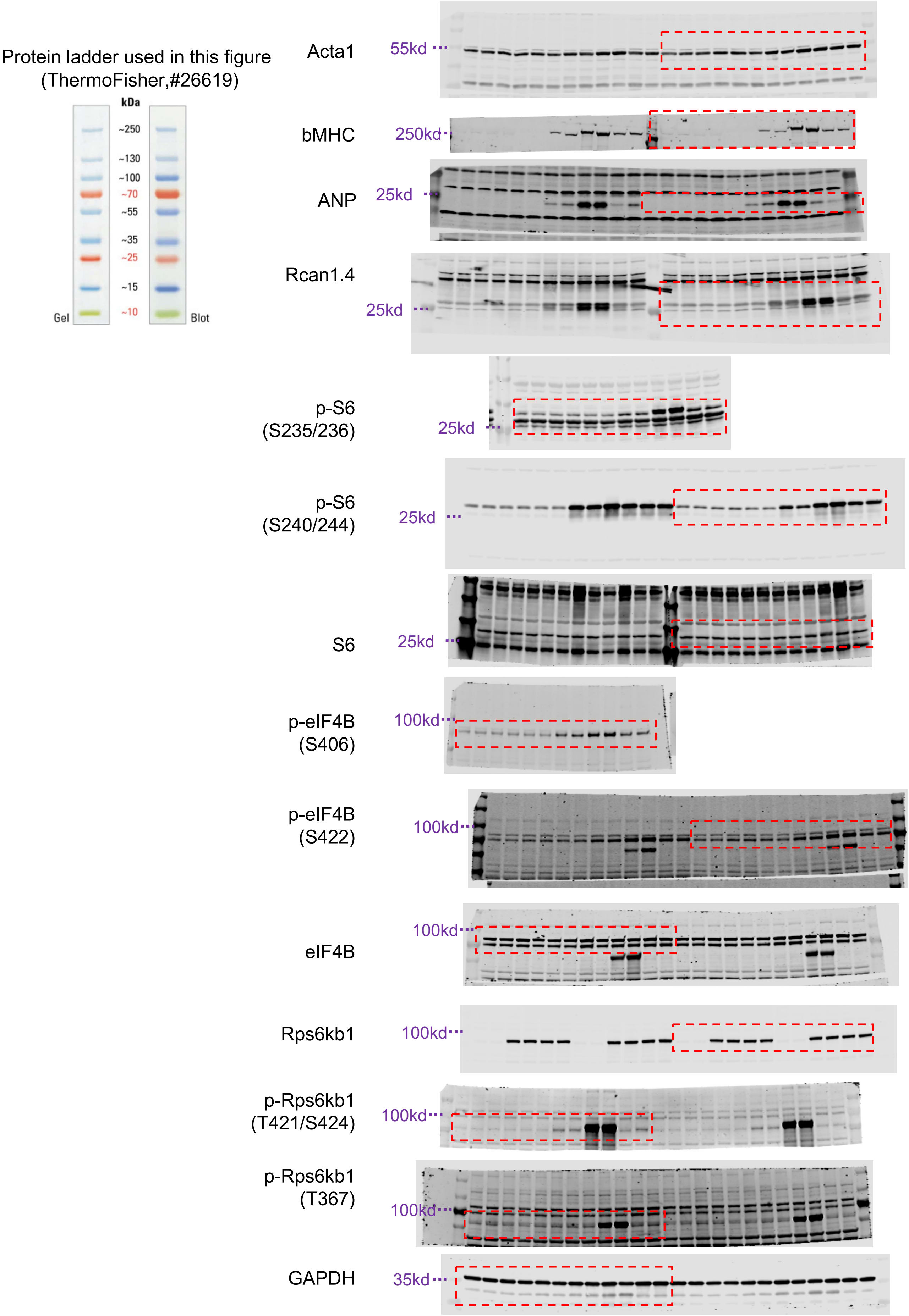

Supplement: Unedited blot and gel images [file jciinsight-11-190760-s025.pdf]
