# Supplementary material for: Phosphoproteomics identification of ERK-dependent activation of Rps6kb1 in cardiac hypertrophy
Source: JCI Insight. 2026 Jan 8;11(4):e190760. doi: 10.1172/jci.insight.190760 (PMC12956007; doi:10.1172/jci.insight.190760)
Supplement: Supplemental data [file jciinsight-11-190760-s026.pdf]

Supplemental Information

Chao Li[1,2,#], Pengfei Zhang[3,#], Kai Zhang[3], Jane A. Cook[1], Weidan Song[1], Megan Virostek[2], Lily A. Slotabec[4], Nadiyeh Rouhi[4], Mohammed Hazari[2], Michael I. Adenawoola[4], Xiaofei Liu[5], Hao Zhang[5], Guangyu Zhang[1], Erica L. Niewold[1], Qinfeng Li[1], Yong Fang[1], Waleed M. Elhelaly[1], Xue-Nan Sun[2], Xuejiang Guo[5], Andrew Lemoff[6], Yingfeng Deng[3], Thomas G. Gillette[1], Ji Li[4,7], Philipp E. Scherer[2], Zhao V. Wang[3,*]

[1] Division of Cardiology, Department of Internal Medicine, The University of Texas Southwestern Medical Center, Dallas, TX 75390, USA.

[2] Touchstone Diabetes Center, The University of Texas Southwestern Medical Center, Dallas, TX 75390, USA.

[3] Department of Diabetes and Cancer Metabolism, Beckman Research Institute, City of Hope National Medical Center, Duarte, CA 91010, USA.

[4] Department of Physiology and Biophysics, University of Mississippi Medical Center, Jackson, MS 39216, USA.

[5] State Key Laboratory of Reproductive Medicine and Offspring Health, Department of Histology and Embryology, Nanjing Medical University, Nanjing, China.

[6] Department of Biochemistry, The University of Texas Southwestern Medical Center, Dallas, TX 75390, USA.

[7] G.V. (Sonny) Montgomery VA Medical Center, Jackson, MS 39216, USA

[#] Chao Li and Pengfei Zhang contributed equally to this study.

*** Address for correspondence:**

Zhao V. Wang, PhD

1500 East Duarte Road, Fox North Building, Department of Diabetes and Cancer Metabolism, Beckman Research Institute, City of Hope National Medical Center, Duarte, CA 91010, USA

Tel: 1-626-218-6565

Email: zhaowang@coh.org

Supplemental Materials and Methods

Animals

All animal procedures were approved by the Institutional Animal Care and Use Committee of the University of Texas Southwestern Medical Center (UTSW) and City of Hope National Medical Center (COH) and conform to the NIH Guide for the Care and Use of Laboratory Animals. Mice were bred into the C57BL/6N background and maintained at a light/dark cycle of 12/12 hours in a mouse facility with temperature control. All mice had free access to water and chow food (Teklad, #2916). For animal surgeries, mice were anesthetized by a cocktail of ketamine (100 mg/kg, intraperitoneal injection) and xylazine (5 mg/kg, intraperitoneal injection) 30 minutes before operation. Mice were gently restrained and deeply anesthetized with pentobarbital (100 mg/kg, intraperitoneal injection) at the termination of experiments.

Generation of the cardiac specific Rps6kb1 knockout mouse model was achieved by crossing Rps6kb1$^{F/F}$ mice (1) with αMHC-Cre transgenic animals.

To obtain cardiomyocyte-specific, inducible overexpression of Rps6kb1 (wild-type or 4A mutant), we generated transgenic mouse models for C-terminal HA-tagged Rps6kb1 under the control of 7 tetracycline responsive elements (TRE). The pTRE vector includes a rabbit β-globin 3'-UTR (Clontech Laboratories, Takara) to enhance translational efficiency. TRE-Rps6kb1 plasmid was linearized by digestion, and the appropriate fragment was purified for pro-nuclear injection by the Transgenic Core of the City of Hope National Medical Center. Multiple founders were obtained and screened for the transgene TRE-Rps6kb1. We then crossed positive lines with αMHC-tTA transgenic mice (2). Doxycycline-containing water (0.1 mg/L) was used to inhibit transgene Rps6kb1 expression ("tet-off") in the double transgenic mice during breeding, pregnancy, and weaning. Induction of Rps6kb1 overexpression in cardiomyocytes in the heart was achieved by replacing doxycycline-containing water with regular drinking water for 2–4 weeks. Single transgenic mice, αMHC-tTA or TRE-Rps6kb1, were used as controls.

Transverse aortic constriction (TAC) surgery

TAC surgery was conducted as previous reported (3-5). Briefly, anesthesia was achieved by ketamine (100 mg/kg, I.P.) plus xylazine (5 mg/kg, I.P.). Skin was disinfected with surgical iodine using standard surgical sterile preparative techniques. The appropriate plane of anesthesia was determined based on observations of spontaneous respiration and pain reflexes (toe pinch). Loss of toe pinch reflex was taken to indicate anesthesia. All instruments, supplies, and suture material were sterilized. Intubation was achieved orally, and a small incision in the anterior neck was made to reveal the trachea, facilitating cannulation. Animals were sometimes ventilated at a rate of 120 breaths per minute and at a tidal volume of 0.1 mL. Respiratory rate, body temperature, and heart rate were monitored continuously during surgery. A warming light was used to maintain body temperature within the range of 34°C to 37°C. The aortic arch was accessed via left lateral thoracotomy. A 27G needle was used to ligate the aorta by 6-0 silk suture between the innominate and left carotid arteries and an overlying needle. After ligation, the needle was removed, immediately leaving a discrete region of stenosis in the aorta. The chest was then closed, and animals were observed during recovery from anesthesia. Sham mice underwent the same procedure without constriction. Surgeons were blinded to treatment/genotype groups.

Echocardiography

Unconstrained, conscious mice were used to evaluate cardiac function by echocardiography (Visual Sonics, #Vevo 3100, MS400C probe) as previously described (6). M-mode images of the short-axis view at the level of papillary muscles were captured and analyzed. Heart rate was also recorded. Left ventricular internal diameters at end diastole (LVID, diastolic) and at end systole

(LVID, systolic) were determined based on M-mode recordings. Fractional shortening = ([LVID, diastolic - LVID, systolic]/LVID, diastolic) × 100%.

Chemicals

The following chemicals and reagents were used: phenylephrine (Sigma, #P6126), U0126 (Cayman, #70970), PD0325901 (Cayman, #13034), Rapamycin (Sigma, #553210), KN93 (Cayman, #13319), Bisindolylmaleimide I (BIM, Cayman, #13298), H89 (Cayman, # 10010556), LY29400(Cayman, #70920), and AKT8 (Sigma, #124018).

Plasmids

HA-Rps6kb1 plasmid (#8984) were purchased from Addgene. The 1A, 3A, and 4A mutations of HA-Rps6kb1 were generated by the Q5® Site-Directed Mutagenesis Kit (NEB, #E0554S). Expression plasmids for myc-ERK2-MEK1-LA were kindly provided by Melanie Cobb, PhD (the University of Texas Southwestern Medical Center, Dallas, Texas, USA) (7).

AAV9 packaging and in vivo delivery

Myc-ERK2-MEK1-LA and HA-Rps6kb1 were clone into pAAV:cTNT::Luciferase (Addgene, #69915). AAV9 viruses were packaged in Boston Children's Hospital Viral Core and injected into newborn mice (day 3–4) at the dose of $1x10^{11}$ VP/gram body weight as previously described (8, 9).

Neonatal rat ventricular myocyte (NRVM) isolation and treatment

NRVMs were isolated from ventricles of 1 to 2 days old Sprague-Dawley rats (Charles River Laboratories) using the Cardiomyocyte Isolation Kit (Cellutron, #NC-6031) as previously performed (4). Briefly, single-cell suspension digested from ventricles was pre-plated twice for 2 hours to remove neonatal fibroblasts. Next, NRVMs were plated at a density of 1,250 cells/mm^2 in plating medium (DMEM/M199 = 3:1) with 5% fetal bovine serum (FBS), 10% horse serum, 1% penicillin/streptomycin, and bromodeoxyuridine (BrdU, 100 μM). After 24 hours, cells were washed with PBS and cultured in reduced-serum medium (DMEM/M199 = 3:1, 1% FBS, 1% penicillin/streptomycin, and 100 μM BrdU). After another 24 hours, NRVMs were switched to serum-free medium (DMEM/M199 = 3:1, 1% penicillin/streptomycin). Cells were then used for various treatments, including siRNA knockdown (Sigma, #SASI_Rn01_00107756, SASI_Rn01_00107757 against Rps6kb1), adenovirus infection, and hypertrophy stimulation.

^{3}H-leucine incorporation assay

NRVMs were cultured with serum-free medium in 6-well plates. After 24 hours, Rps6kb1 was silenced by siRNA. L-[3,4,5-^{3}H]-leucine (PerkinElmer, #NET460A001MC, 2 μCi/ml), Adeno-GFP, and Adeno-caMEK1 were added into medium. After another 24 hours, cells were washed 3 times with ice-cold PBS and incubated with 2 mL trichloroacetic acid (LabChem, #LC262302, 10%) for 30 minutes at 4°C with gentle agitation. After 2 washes with ice-cold 95% ethanol, samples were incubated with 1 mL NaOH (0.5 N) at 37°C for 18–24 hours with gentle agitation. Finally, samples were neutralized with 1 mL HCl (0.5 N), and all contents were transferred to scintillation vials. After mixing with scintillation solution (MP Biomedicals, EcoLite, #882475, 18 mL), radioactivity was detected by a scintillation counter (Beckman, #LS5000TA).

Western blotting

Total proteins were prepared from cells or cardiac tissues using RIPA lysis and extraction buffer (Thermo Fisher Scientific, #89900), supplemented with protease and phosphatase inhibitors (Thermo Fisher Scientific, #88669). Protein concentration was quantified with BCA kits (Thermo Fisher Scientific, #23225). Equal total proteins of each sample were loaded on 26-well Criterion

TGX precast gels (Bio-Rad, 4-20%, #5671095) and transferred onto nitrocellulose membranes (Bio-Rad, #1704157). After blocking with 5% non-fat milk or 3% BSA for 1 hour at room temperature, membranes were incubated with primary antibodies overnight at 4°C, followed by incubation with secondary antibodies for 1 hour and imaging with an Odyssey scanner (Li-Cor). The following antibodies were used: GAPDH (Fitzgerald, #10R-G109A), Rcan1 (Sigma, #D6694), ERK1/2 (Cell Signaling, #4696), p-ERK1/2 T202/Y204 (Cell Signaling, #9101), Rps6kb1 (Cell Signaling, #2708), p-Rps6kb1 T389 (Cell Signaling, #9206), p-Rps6kb1 T421/S424 (Cell Signaling, #9204), S6 (Cell Signaling, #2317), p-S6 S240/S244 (Cell Signaling, #5364), p-S6 S235/S236 (Cell Signaling, #4858), 4EBP1 (Cell Signaling, #9644), p-4EBP1 T37/T46 (Cell Signaling, #2855), Myc (Santa Cruz, #sc-40), HA (Cell Signaling, #3724), eIF4B (Cell Signaling, #3592), p-eIF4B S422 (Cell Signaling, #3591), p-eIF4B S406 (Cell Signaling, #5399), Acta1 (Sigma, #A2066), βMHC (Sigma, #M8421), ANP (Proteintech, #27426-1-AP), BNP (Abcam, #ab19645), GST (Cell Signaling, #2622), IRDye 800 CW goat anti-rabbit secondary antibody (Li-Cor, #925-32211), and Alexa Fluor 680-conjugated anti-mouse secondary antibody (Thermo Fisher Scientific, #A21057). Specific polyclonal antibodies against Rps6kb1 phospho-T367 (synthetic peptide FTRQ[pT]PVDS) were produced by Abclonal.

Rps6kb1 activity assay

For Rps6kb1 activity measurement, the expression plasmid HA-Rps6kb1 or its mutant variants were transfected into HEK293A cells (Thermo Fisher Scientific, #R70507) with or without the ERK2-MEK1-LA plasmid. After two days, HA-Rps6kb1 was enriched by immunoprecipitation. The activity of Rps6kb1 was then quantified using the p70 S6K Activity Kit (Enzo, #ADI-EKS-470).

Co-immunoprecipitation

For co-immunoprecipitation (co-IP), cells were lysed in IP buffer (20 mM Tris, 100 mM NaCl, 1 mM EDTA-2Na, 10% glycerol, 0.1% NP-40, 1% Triton X-100, pH 7.2) with protease and phosphatase inhibitors (Thermo Fisher Scientific, #88669). Protein lysates were centrifuged at 11,000 *g* for 10 minutes at 4°C. Supernatant was incubated with magnetic beads (Thermo Fisher Scientific, #78609) for 1 hour at 4°C to eliminate non-specific binding proteins. Then, antibodies and magnetic beads were added to lysate, and the mixture was rotated at 4°C overnight. Beads were next washed with IP buffer for 4 times and boiled with 2 x SDS-PAGE loading buffer for 10 minutes.

In vitro kinase assay and GST pulldown

For in vitro kinase assay, purified Rps6kb1 protein (Creative Biomart, #RPS6KB1-29261TH) was incubated with or without ERK2 protein (Abcam, #ab155812) in protein kinase assay buffer (50 mM Tris-HCl, 10 mM $MgCl_2$, 0.1 mM EDTA, 2 mM DTT, 0.01% Brij, pH 7.5) for 20 minutes at 30°C. Then, samples were boiled with loading buffer for western blotting.

For GST pulldown, purified His-Rps6kb1 protein was incubated with GST-ERK2 or GST protein in IP buffer (20 mM Tris, 100 mM NaCl, 1 mM EDTA-2Na, 10% glycerol, 0.1% NP-40, 1% Triton X-100, pH 7.2) with protease and phosphatase inhibitors overnight at 4°C. Then, Glutathione Magnetic Agarose Beads (Thermo Fisher Scientific, #78601) were added into samples for 2 hours. Beads were next washed with IP buffer for 4 times and boiled with 2 x SDS-PAGE loading buffer for 10 minutes.

Proteomics

For phosphoproteomics, NRVMs were treated with vehicle or phenylephrine (PE, 50 μM) for 30 minutes. Total proteins were extracted. Equal total proteins (1 mg for each treatment) were submitted to the Proteomics Core Facility of UTSW. Phospho-peptide enrichment was conducted by the Core, followed by LC-MS/MS. Briefly, samples were reconstituted in 50 mM

triethylammonium bicarbonate (TEAB), 5% SDS. Tris (2-carboxyethyl) phosphine (TCEP) was added to a final concentration of 10 mM and incubated at 56°C for 30 minutes, followed by the addition of iodoacetamide to a final concentration of 20 mM, which was incubated at room temperature for 30 minutes in dark. Solutions were then acidified with 12% phosphoric acid, followed by the addition of 6 times the volume of S-Trap binding buffer (90% methanol, 10% 1 M TEAB), and then loaded onto the S-Trap column (Protifi). The column was washed 3 times with 150 μL of binding buffer followed by the addition of trypsin (1:10) in 50 mM TEAB and overnight incubation at 37°C. Peptides were recovered by eluting the S-Trap column with 35 μL of 50 mM TEAB, 0.2% formic acid (FA), and 50% ACN in 0.2% FA, sequentially. The eluate containing the peptides was dried and then cleaned using a 96-well Waters Oasis HLB solid-phase extraction plate.

Peptide samples were reconstituted with the binding buffer from the Thermo High-Select TiO_2 Phosphopeptide Enrichment kit. The pH of the samples was verified to be less than 3 using pH paper and then loaded to TiO_2 Spin Tips for binding and washing. The flowthrough was collected for secondary enrichment with High-Select Fe-NTA Phosphopeptide enrichment columns (Thermo Fisher Scientific). The phosphopeptides collected from each enrichment step were combined, dried, and reconstituted in 2% (v/v) ACN, 0.1% trifluoroacetic acid in water. The unphosphorylated peptide fractions from each step were prepared the same way for analysis.

All samples were injected onto an Orbitrap Fusion Lumos mass spectrometer coupled to an Ultimate 3000 RSLC-Nano liquid chromatography system. Samples were injected onto a 75 μm i.d., 75-cm long EasySpray column (Thermo Fisher Scientific) and eluted with a gradient from 0–28% buffer B over 90 minutes. Buffer A contained 2% (v/v) ACN and 0.1% formic acid in water, and buffer B contained 80% (v/v) ACN, 10% (v/v) trifluoroethanol, and 0.1% formic acid in water. The mass spectrometer operated in positive ion mode with a source voltage of 1.5 kV and an ion transfer tube temperature of 275°C. MS scans were acquired at 120,000 resolution in the Orbitrap and, up to 10 MS/MS spectra were obtained in the ion trap for each full spectrum acquired using higher-energy collisional dissociation (HCD) for ions with charges 2–7. Dynamic exclusion was set for 25 seconds after an ion was selected for fragmentation.

Raw MS data files were analyzed using Proteome Discoverer v3.0 (Thermo Fisher Scientific), with peptide identification performed using Sequest HT searching against the rat reviewed protein database from UniProt. Fragment and precursor tolerances of 10 ppm and 0.6 Da were specified, and three missed cleavages were allowed. Carbamidomethylation of Cys was set as a fixed modification, with oxidation of Met and phosphorylation of Ser, Thr, and Tyr set as variable modifications. The false-discovery rate (FDR) cutoff was 1% for all peptides. Intensities were used for quantification of phosphorylation sites. To explore kinase-substrate relationships identified by the phosphoproteomics analyses, motifs enriched in differential phosphorylation sites (fold change > 2) were analyzed using MOMO (10) with default parameters, and only those with matching phosphorylation sites upregulated after treatment (paired Student's *t* test with Benjamini–Hochberg correction, FDR-q < 0.05) were considered significant. The kinase-motif-substrate network was constructed using Cytoscape (version 3.7.1) (11). Gene ontology (GO) enrichment and KEGG pathway analyses were performed using clusterProfiler (12) and the org.Rn.eg.db package. The sequence logo was obtained using the ggseqlogo package in R.

To identify new phosphorylation sites of Rps6kb1, we transfected HA-tagged Rps6kb1 plasmids along with constitutively active ERK2-MEK1-LA plasmids into HEK293A cells. We next treated the cells with rapamycin (20 nM) to inhibit the mTORC1 signaling. After immunoprecipitation with HA antibody, Rps6kb1 was separated on an SDS-PAGE gel first for Coomassie blue staining. The gel slice containing Rps6kb1 was then cut and minced for MS by the Proteomics Core of UTSW.

Histology

Mouse hearts were excised and fixed in 10% neutralized formalin for 3 days at 4°C with gentle agitation. Paraffin sections of 5-μm thickness were used for hematoxylin and eosin (H&E) staining and Masson's trichrome staining in the Molecular Pathology Core of UTSW or City of Hope National Medical Center. For wheat germ agglutin (WGA) staining, heart sections were first deparaffinized and then rehydrated. After blocking with 5% normal goat serum in PBS for 1 hour, sections were incubated in blocking buffer (5% normal goat serum, 1% BSA in PBS) with Alexa Fluor 594-conjugated WGA (Thermo Fisher Scientific, #W11262, 10 μg/mL) overnight at 4°C. After 3 washes with PBS, sections were mounted with DAPI (Sigma, #DUO82040) for imaging and quantification of cardiomyocyte cross-sectional area.

Statistical analysis

Data are represented as mean±SEM. Normality of data distribution was evaluated by using Shapiro-Wilk test. Two-tailed Student's *t* test was performed to compare differences between two groups. For multiple group comparisons with 1 variable, one-way ANOVA was conducted, followed by Tukey's multiple comparison test. For multiple group comparisons with more than 2 variables, two-way ANOVA was conducted, followed by Tukey's multiple comparison test. A p value of < 0.05 was considered statistically significant. Statistical analyses were performed using Graphpad Prism software 8.4.2.

Supplemental References

1. Ito TK, Lu C, Khan J, Nguyen Q, Huang HZ, Kim D, et al. Hepatic S6K1 partially regulates lifespan of mice with mitochondrial complex I deficiency. *Front Genet.* 2017;8:113.
2. Yu Z, Redfern CS, and Fishman GI. Conditional transgene expression in the heart. *Circ Res.* 1996;79(4):691-697.
3. Wang X, Deng Y, Zhang G, Li C, Ding G, May HI, et al. Spliced X-box binding protein 1 stimulates adaptive growth through activation of mTOR. *Circulation.* 2019;140(7):566-579.
4. Tran DH, May HI, Li Q, Luo X, Huang J, Zhang G, et al. Chronic activation of hexosamine biosynthesis in the heart triggers pathological cardiac remodeling. *Nat Commun.* 2020;11(1):1771.
5. Zhang G, Wang X, Bi X, Li C, Deng Y, Al-Hashimi AA, et al. GRP78 (glucose-regulated protein of 78 kDa) promotes cardiomyocyte growth through activation of GATA4 (GATA-binding protein 4). *Hypertension.* 2019;73(2):390-398.
6. Wang ZV, Deng Y, Gao N, Pedrozo Z, Li DL, Morales CR, et al. Spliced X-box binding protein 1 couples the unfolded protein response to hexosamine biosynthetic pathway. *Cell.* 2014;156(6):1179-1192.
7. Robinson MJ, Stippec SA, Goldsmith E, White MA, and Cobb MH. A constitutively active and nuclear form of the MAP kinase ERK2 is sufficient for neurite outgrowth and cell transformation. *Curr Biol.* 1998;8(21):1141-1150.
8. Li C, Sun XN, Chen BY, Zeng MR, Du LJ, Liu T, et al. Nuclear receptor corepressor 1 represses cardiac hypertrophy. *EMBO Mol Med.* 2019;11(11):e9127.
9. Prendiville TW, Guo H, Lin Z, Zhou P, Stevens SM, He A, et al. Novel roles of GATA4/6 in the postnatal heart identified through temporally controlled, cardiomyocyte-specific gene inactivation by adeno-associated virus delivery of Cre recombinase. *PLoS One.* 2015;10(5):e0128105.
10. Cheng A, Grant CE, Noble WS, and Bailey TL. MoMo: discovery of statistically significant post-translational modification motifs. *Bioinformatics.* 2019;35(16):2774-2782.

11. Shannon P, Markiel A, Ozier O, Baliga NS, Wang JT, Ramage D, et al. Cytoscape: a software environment for integrated models of biomolecular interaction networks. *Genome Res.* 2003;13(11):2498-2504.
12. Yu G, Wang LG, Han Y, and He QY. clusterProfiler: an R package for comparing biological themes among gene clusters. *OMICS.* 2012;16(5):284-287.

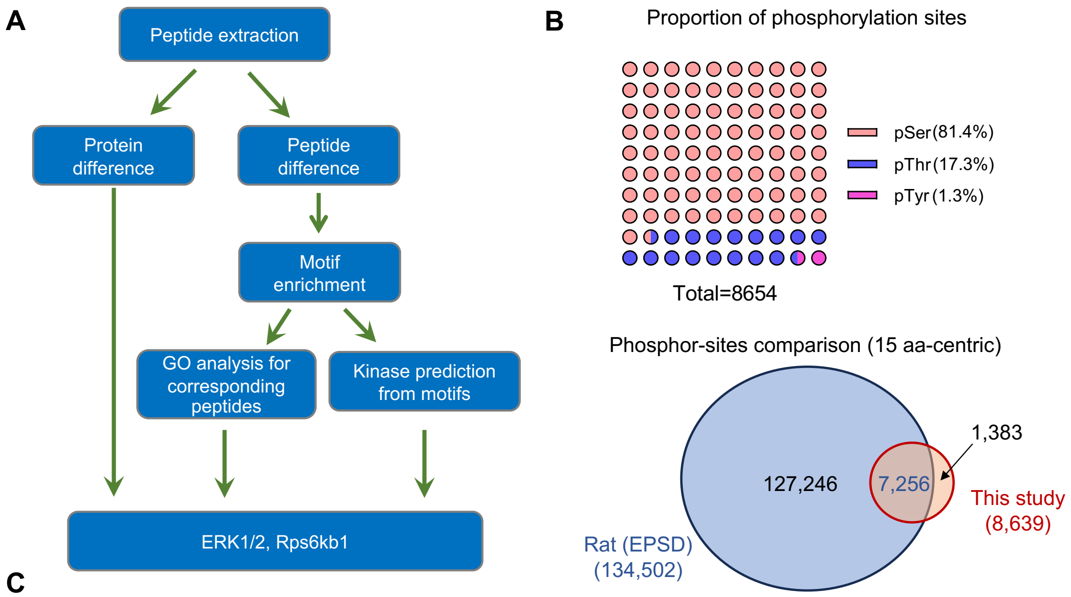

C

Motif	#MatchedSites_Diff	#MatchedProteins_Diff	#MatchedSites_All	p.value(Pair TTest)	p.adjust	Matched Kinase
RRx[S]	92	89	185	8.191E-09	8.191E-08	PKA kinase substrate motif
[S]P	734	497	2059	3.741E-04	1.870E-03	GSK-3, ERK1, ERK2, CDK5 substrate motif
Px[S]P	170	147	424	2.057E-03	5.143E-03	ERK1, ERK2 Kinase substrate motif
RKx[S]	57	54	105	1.999E-03	5.143E-03	PKG Kinase substrate motif
RxRxx[S]	86	76	236	1.408E-02	2.817E-02	AKT Kinase substrate motif; RPS6K Kinase substrate motif (RPS6KA1/2/3&RPS6KB1)

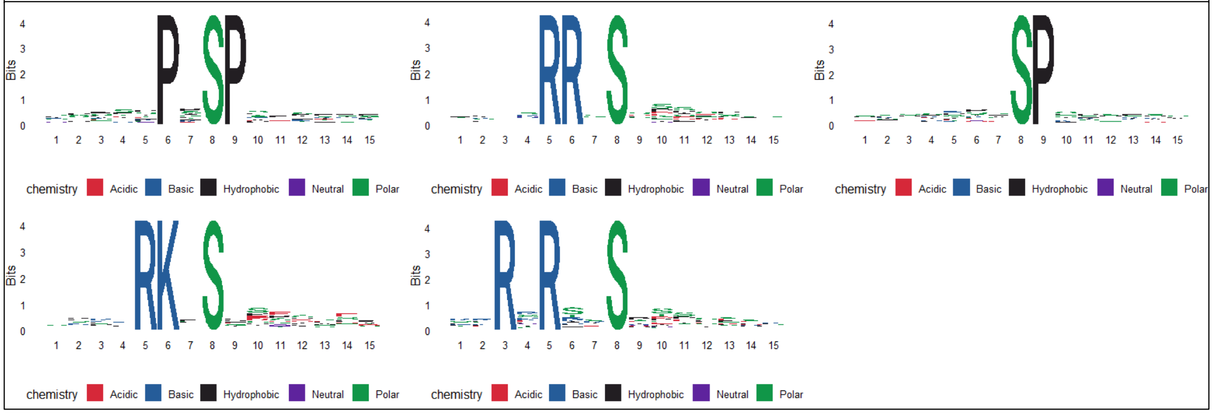

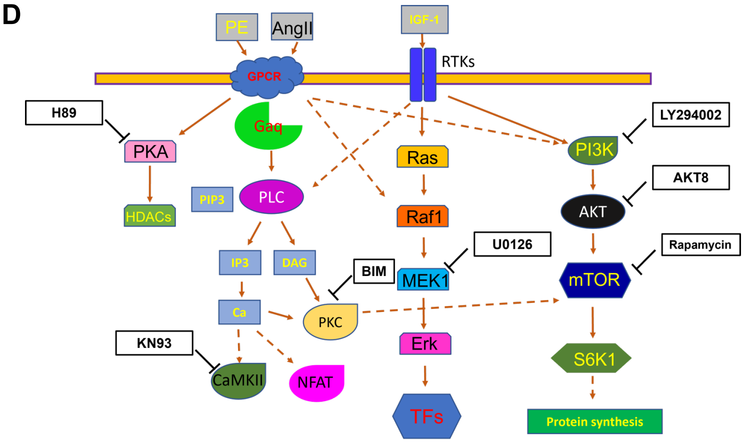

Supplemental Figure 1. Phosphoproteomics in primary cardiomyocyte under growth stimulation.

A. Workflow for the phosphoproteomics experiment.

B. Distribution of phosphorylation sites.

C. Five motifs were enriched.

D. The phosphorylation network in cardiac hypertrophy. Seven kinase inhibitors were tested in this study, and their targets were labelled.

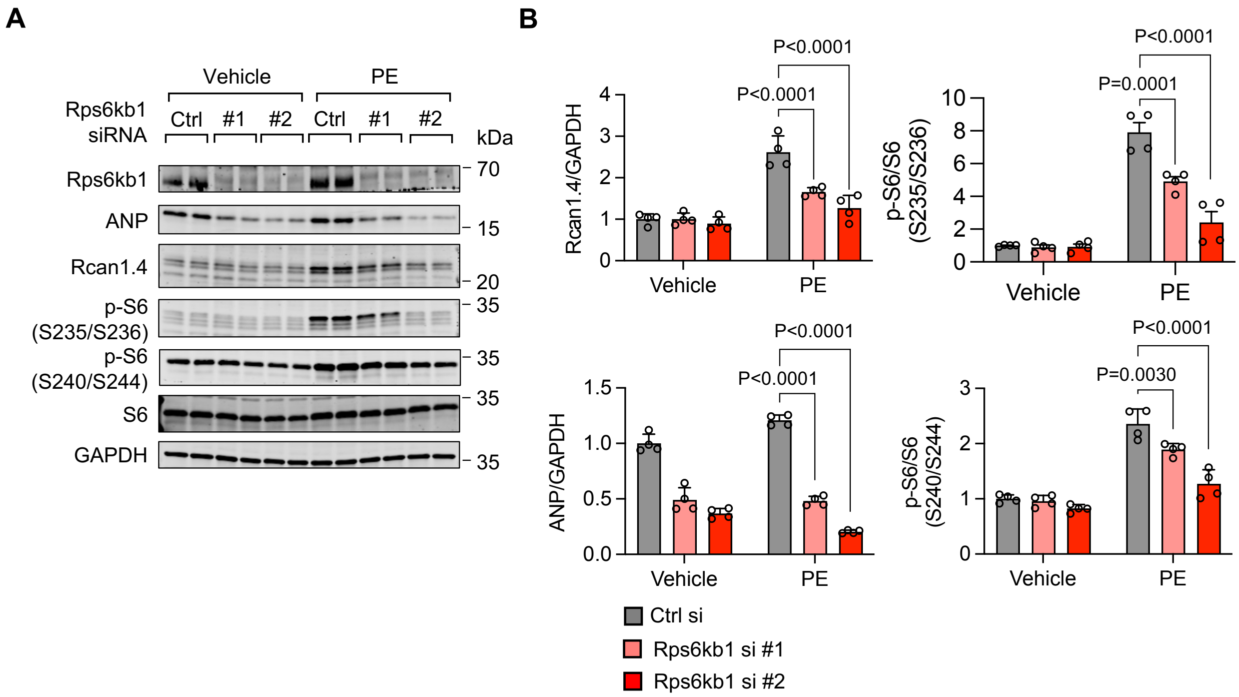

Supplemental Figure 2. Rps6kb1 is required for phenylephrine-induced cardiomyocyte growth in vitro.

- **A.** Rps6kb1 silencing reduced the expression of marker genes related to cardiomyocyte growth at the protein level under phenylephrine (PE) treatment. Two independent siRNAs against Rps6kb1 were used. NRVMs were treated with PE to induce hypertrophy. Western blotting was conducted to examine the phosphorylation of S6 and the expression of ANP and Rcan1.4.
- **B.** Quantification of (**A**). n=4.

Two-way ANOVA was conducted, followed by Tukey's multiple comparisons test for **B**. Data are presented as mean±SEM.

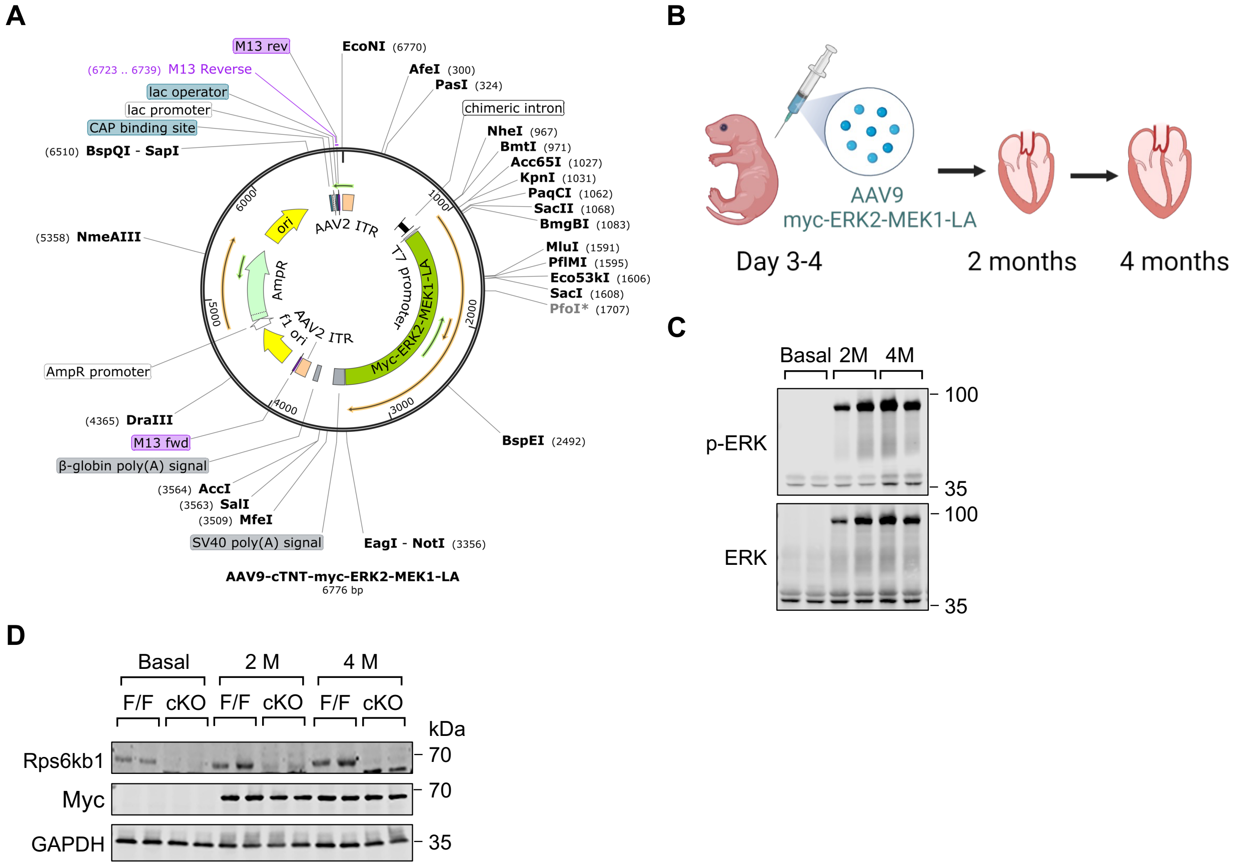

Supplemental Figure 3. Overexpression of myc-ERK2-MEK-LA in mice in vivo.

- **A.** Construct map of AAV9-myc-ERK2-MEK-LA.
- **B.** Constitutively active ERK2 was delivered to cardiomyocytes in the heart through AAV9 infection. Heart tissues were collected 2 and 4 months, respectively, after infection.
- **C.** Western blot analysis for phosphorylated ERK and total ERK (both endogenous and overexpressed) of the heart tissues from control and caMEK1 overexpression mice, respectively.
- **D.** Validation of the cardiac-specific conditional knockout (cKO) mouse model of Rps6kb1 and caMEK1 overexpression in the heart. Note that caMEK1 was tagged by Myc.

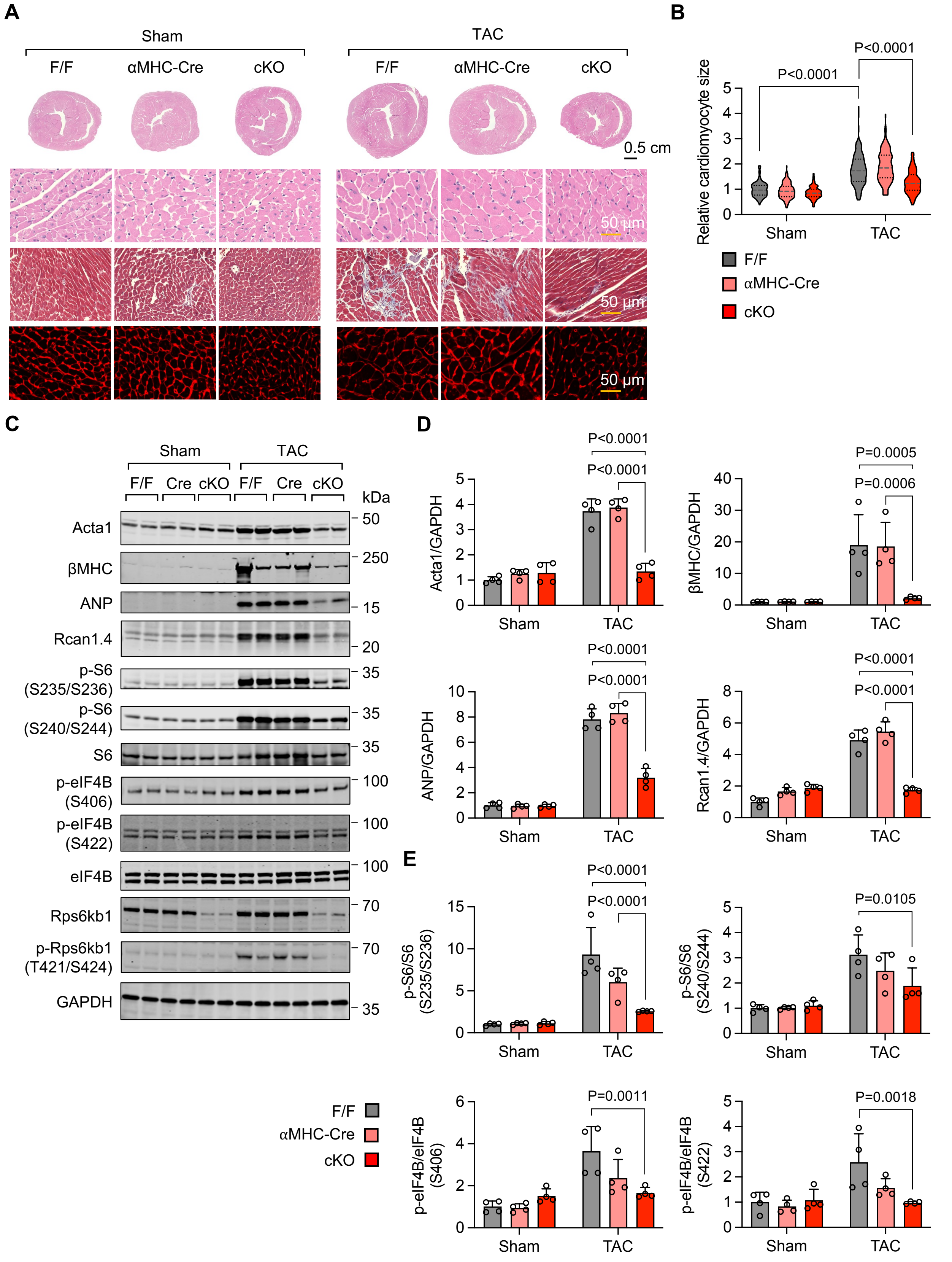

A
Sham
TAC
F/F
αMHC-Cre
cKO
0.5 cm
50 μm
B
Relative cardiomyocyte size
P<0.0001
P<0.0001
F/F
αMHC-Cre
cKO
C
Sham
TAC
F/F
Cre
cKO
kDa
Acta1
βMHC
ANP
Rcan1.4
p-S6 (S235/S236)
p-S6 (S240/S244)
S6
p-eIF4B (S406)
p-eIF4B (S422)
eIF4B
Rps6kb1
p-Rps6kb1 (T421/S424)
GAPDH
50
250
15
20
35
100
70
D
Acta1/GAPDH
βMHC/GAPDH
ANP/GAPDH
Rcan1.4/GAPDH
P<0.0001
P=0.0005
P=0.0006
E
p-S6/S6 (S235/S236)
p-S6/S6 (S240/S244)
p-eIF4B/eIF4B (S406)
p-eIF4B/eIF4B (S422)
P=0.0105
P=0.0011
P=0.0018

Supplemental Figure 4. Rps6kb1 is required for pressure overload-induced cardiac hypertrophy in vivo.

- **A.** Representative histological images for heart sections from Rps6kb1$^{F/F}$, αMHC-Cre, and cKO mice, respectively, subjected to either sham or TAC operations for 4 weeks. Cardiac tissue sections were harvested for H&E staining (upper), Masson's trichrome staining (middle), and wheat germ agglutin staining (WGA, bottom).
- **B.** Cardiomyocyte cross-sectional area was decreased in Rps6kb1 cKO hearts compared to Rps6kb1$^{F/F}$ or αMHC-Cre controls. A total of 103–106 cardiomyocytes for each group was quantified from WGA staining of (**A**).
- **C.** Western blot analysis for heart samples from Rps6kb1$^{F/F}$, αMHC-Cre, and cKO mice subjected to either sham or TAC operations for 4 weeks. Hypertrophy marker proteins were examined. Two Rps6kb1 targets, eIF4B and S6, were evaluated.
- **D.** Quantification of the protein expression levels of Acta1, Rcan1.4, ANP, and βMHC. n=4.
- **E.** Quantification of the phosphorylation of eIF4B and S6. n=4.

Two-way ANOVA was conducted, followed by Tukey's multiple comparisons test for **B**, **D**, and **E**. Data are presented as mean±SEM.

A

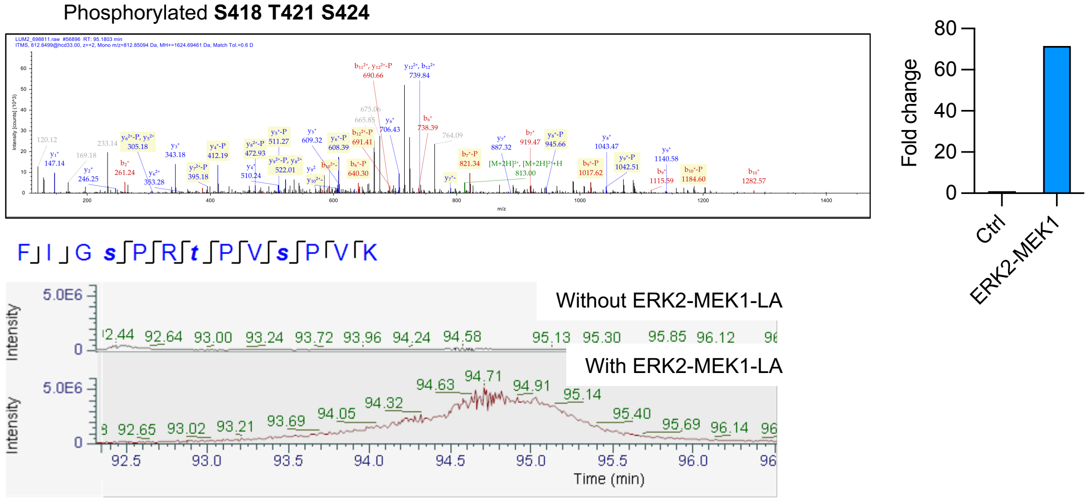

B

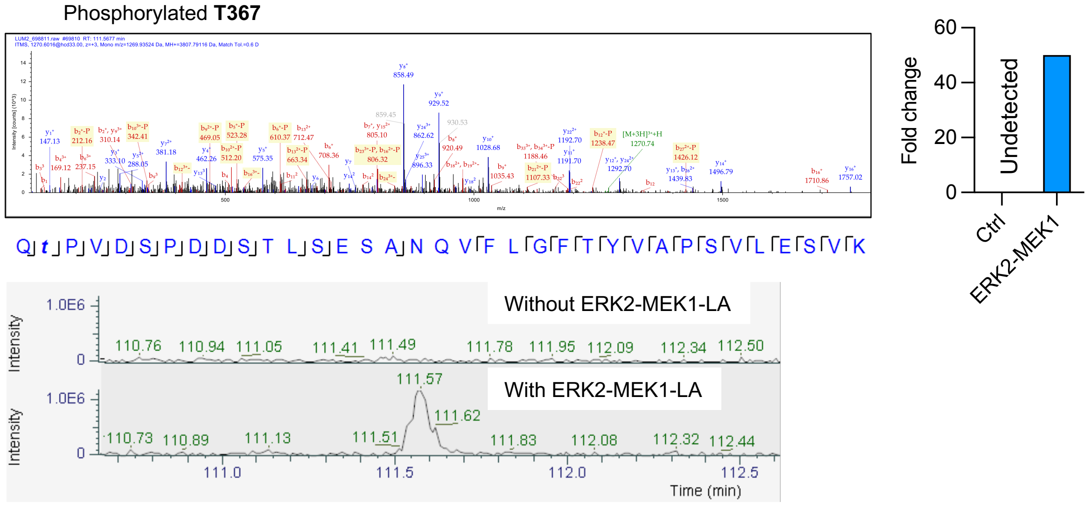

Supplemental Figure 5. Mass spectrometry results for the phosphorylation of Rps6kb1 peptides by ERK.

- **A.** Identification of phosphorylation of Rps6kb1 at the S418/T421/S424 sites by ERK.
- **B.** Identification of phosphorylation of Rps6kb1 at the T367 residue by ERK.

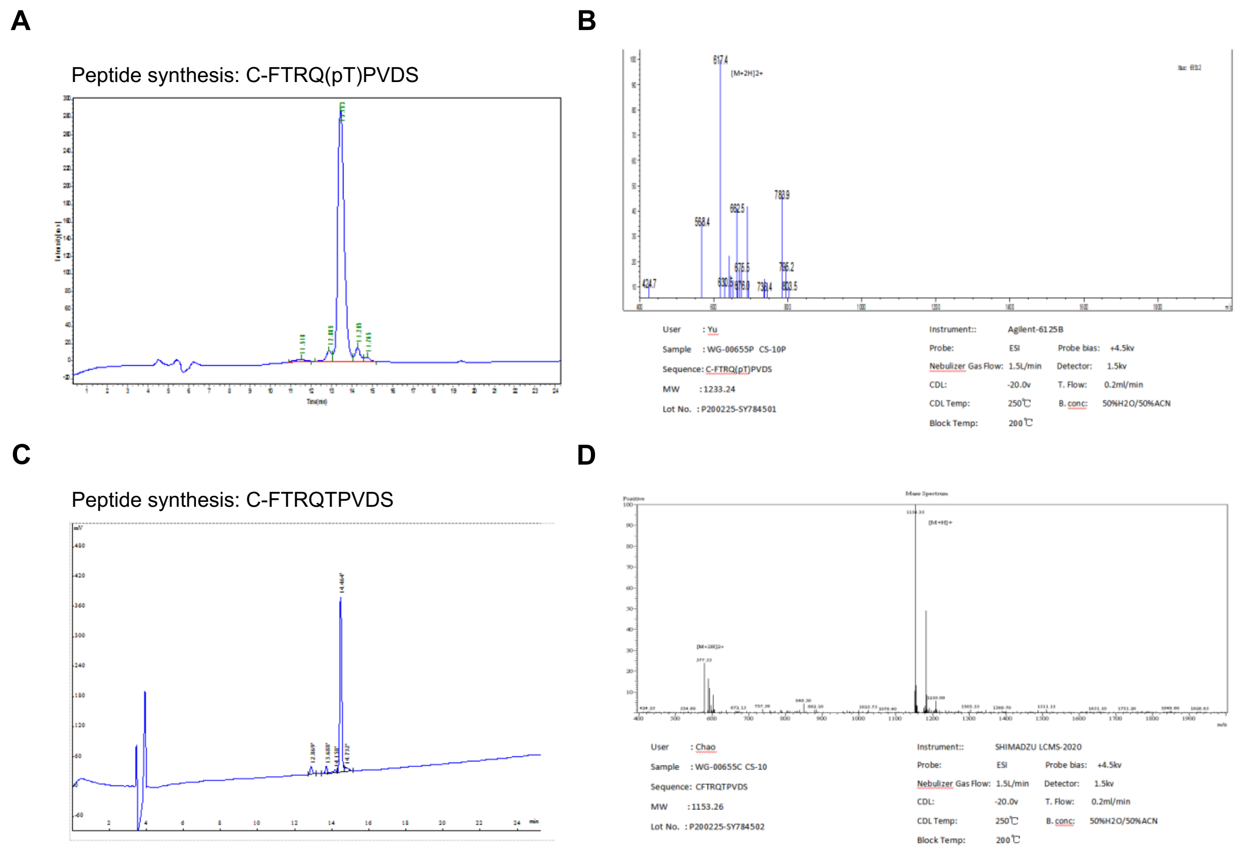

Supplemental Figure 6. Synthesis of peptides of Rps6kb1 around the T367 residue.

- **A.** The peptide FTRQpTPVDS was examined by HPLC.
- **B.** The peptide FTRQpTPVDS was examined by mass spectrometry.
- **C.** The peptide FTRQTPVDS was examined by HPLC.
- **D.** The peptide FTRQTPVDS was examined by mass spectrometry.

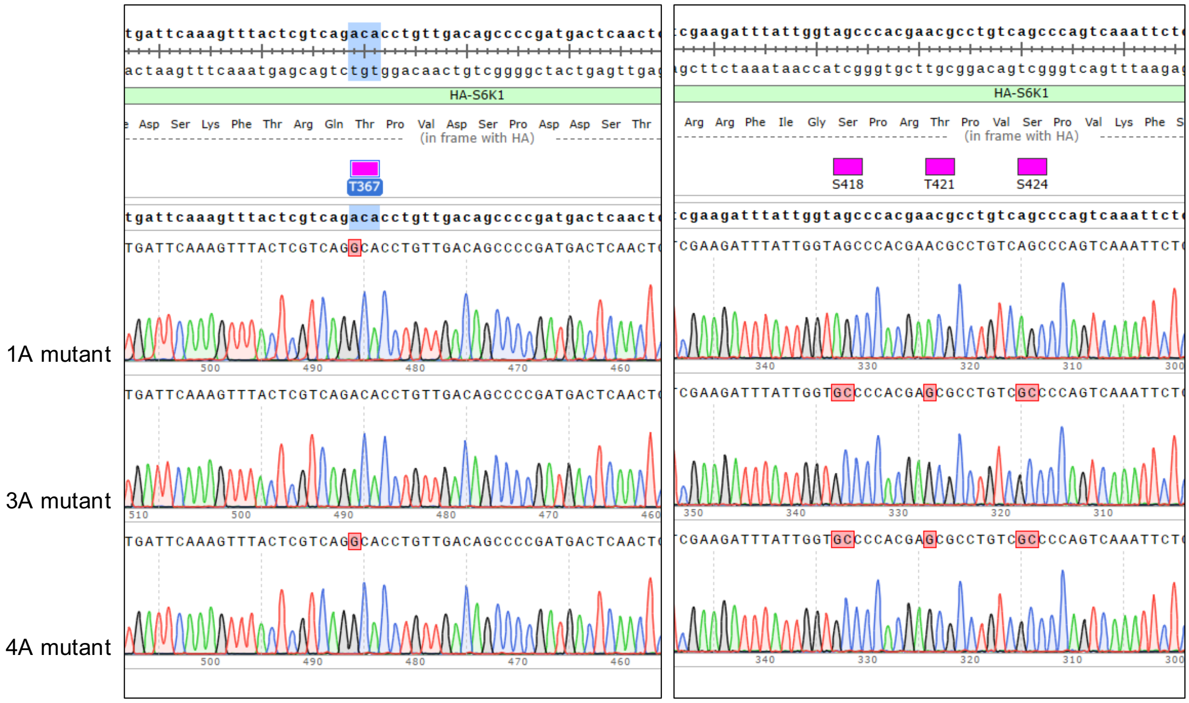

Supplemental Figure 7. Generation of Rps6kb1 point mutations.

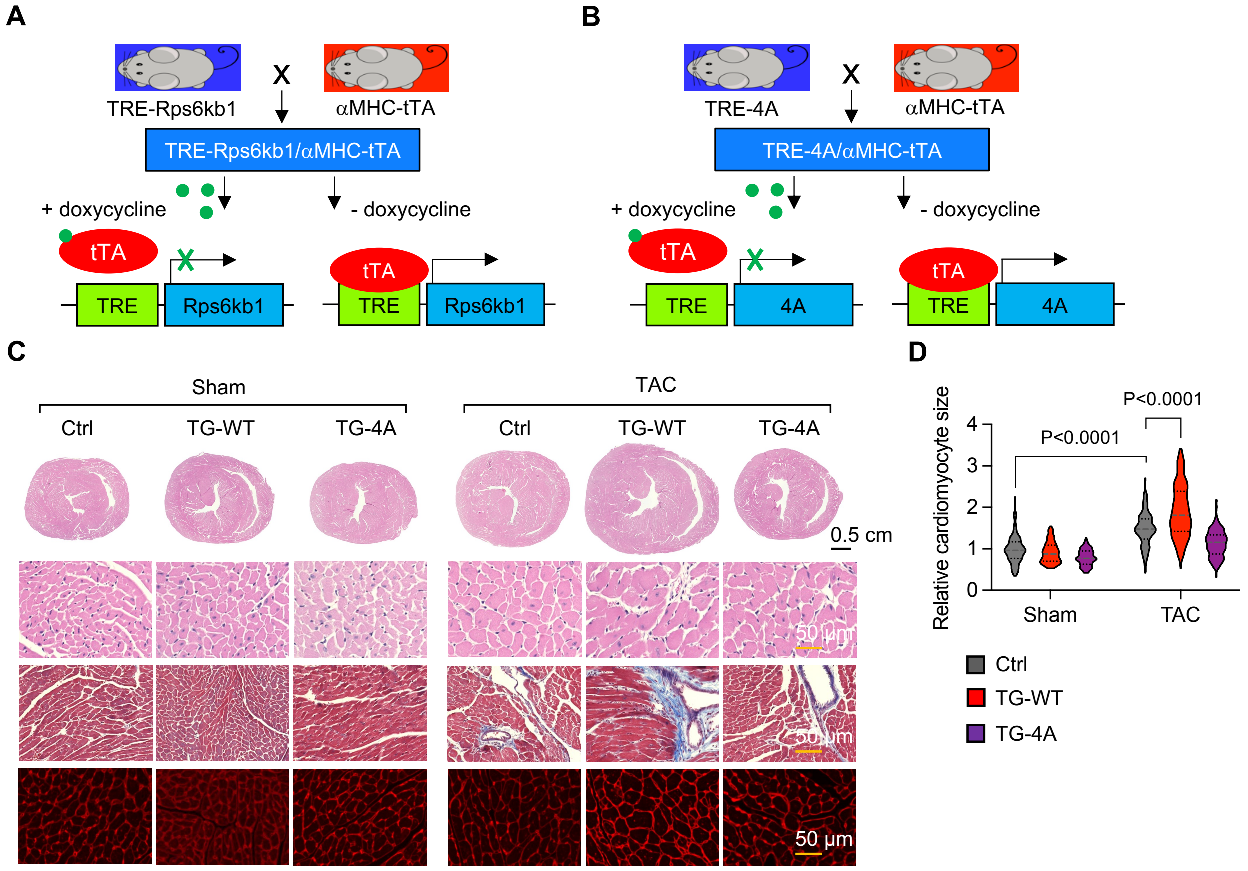

Supplemental Figure 8. Phosphorylation of the T367 and S418/T421/S424 sites is required for the full activation and function of Rps6kb1.

- **A.** Schematic of the cardiomyocyte specific, inducible wild-type Rps6kb1 transgenic mouse model (TG-WT).
- **B.** Schematic of the cardiomyocyte specific, inducible 4A mutant Rps6kb1 transgenic mouse model (TG-4A).
- **C.** Representative histological images for heart sections from control, TG-WT, and TG-4A mice subjected to either sham or TAC operations for 4 weeks. Cardiac tissue sections were harvested for H&E staining (upper), Masson's trichrome staining (middle), and wheat germ agglutin staining (WGA, bottom).
- **D.** Cardiomyocyte cross-sectional area was increased in TG-WT hearts compared to control or TG-4A hearts after TAC. A total of 103–150 cardiomyocytes for each group was quantified from WGA staining of (**D**).

Two-way ANOVA was conducted, followed by Tukey's multiple comparisons test for **D**. Data are presented as mean±SEM.

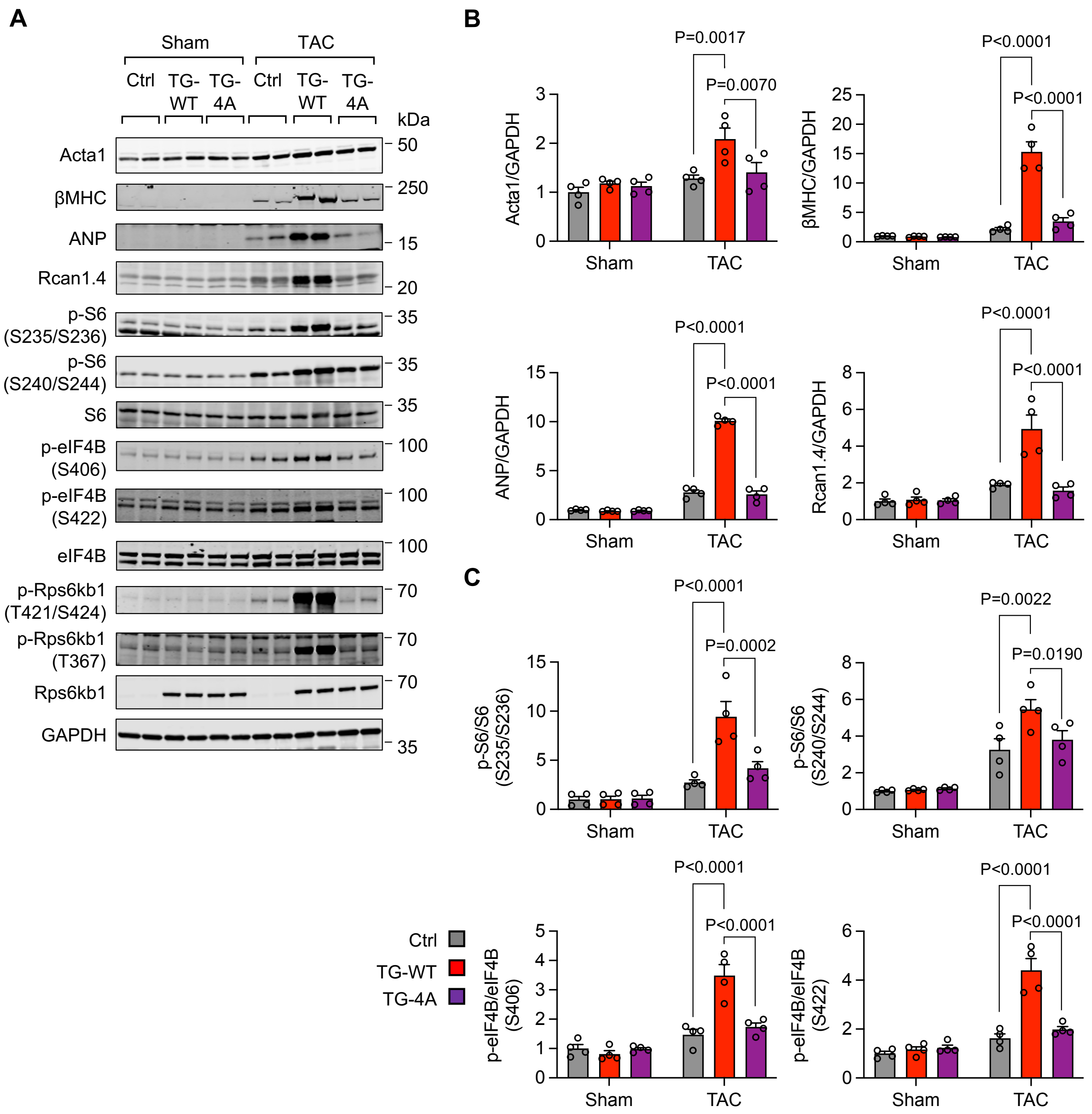

Supplemental Figure 9. The Phosphorylation of T367 and S418/T421/S424 sites of Rps6kb1 is critical for its activity.

- **A.** Western blot analysis of heart samples from control (Ctrl), wild-type Rps6k1 (TG-WT), and 4A mutant Rps6kb1 (TG-4A) transgenic mice subjected to either sham or TAC operations for 4 weeks. Hypertrophy marker proteins were examined. Two Rps6kb1 targets, eIF4B and S6, were evaluated.
- **B.** Quantification of the protein expression levels of Acta1, Rcan1.4, ANP, and βMHC. n=4.
- **C.** Quantification of the phosphorylation of eIF4B and S6. n=4.

Two-way ANOVA was conducted, followed by Tukey's multiple comparisons test for **B** and **C**. Data are presented as mean±SEM.

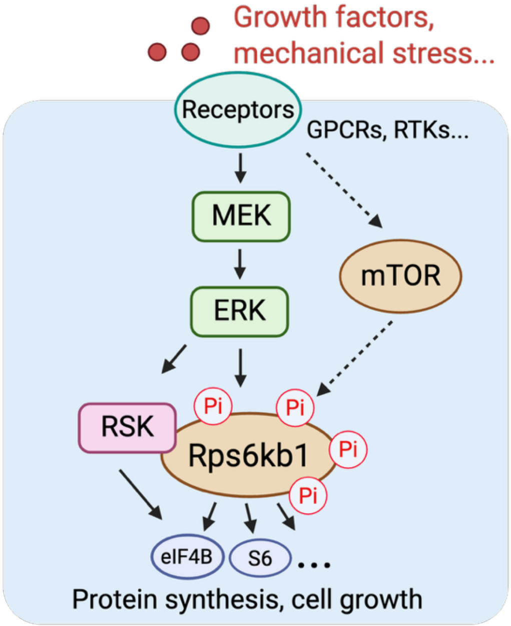

Supplemental Figure 10. The working model.
